# Identification and characterization of small non-coding RNAs from Chinese fir by high throughput sequencing

**DOI:** 10.1186/1471-2229-12-146

**Published:** 2012-08-15

**Authors:** Li-Chuan Wan, Feng Wang, Xiangqian Guo, Shanfa Lu, Zongbo Qiu, Yuanyuan Zhao, Haiyan Zhang, Jinxing Lin

**Affiliations:** 1 Key Laboratory of Plant Molecular Physiology, Institute of Botany, Chinese Academy of Sciences, Beijing, 100093, China; 2 Graduate School of the Chinese Academy of Sciences, Beijing, 100049, China; 3 Bioinformatics Laboratory and National Laboratory of Biomacromolecules, Institute of Biophysics, Chinese Academy of Sciences, Beijing, 100101, China; 4 Medicinal Plant Cultivation Research Center, Institute of Medicinal Plant Development, Chinese Academy of Medical Sciences & Peking Union Medical College, Haidian District, Beijing, 100193, China

**Keywords:** Chinese fir, miRNA, rasiRNA, tasiRNA, *Cunninghamia lanceolata*

## Abstract

**Background:**

Small non-coding RNAs (sRNAs) play key roles in plant development, growth and responses to biotic and abiotic stresses. At least four classes of sRNAs have been well characterized in plants, including repeat-associated siRNAs (rasiRNAs), microRNAs (miRNAs), *trans*-acting siRNAs (tasiRNAs) and natural antisense transcript-derived siRNAs. Chinese fir (*Cunninghamia lanceolata*) is one of the most important coniferous evergreen tree species in China. No sRNA from Chinese fir has been described to date.

**Results:**

To obtain sRNAs in Chinese fir, we sequenced a sRNA library generated from seeds, seedlings, leaves, stems and calli, using Illumina high throughput sequencing technology. A comprehensive set of sRNAs were acquired, including conserved and novel miRNAs, rasiRNAs and tasiRNAs. With BLASTN and MIREAP we identified a total of 115 conserved miRNAs comprising 40 miRNA families and one novel miRNA with precursor sequence. The expressions of 16 conserved and one novel miRNAs and one tasiRNA were detected by RT-PCR. Utilizing real time RT-PCR, we revealed that four conserved and one novel miRNAs displayed developmental stage-specific expression patterns in Chinese fir. In addition, 209 unigenes were predicted to be targets of 30 Chinese fir miRNA families, of which five target genes were experimentally verified by 5' RACE, including a squamosa promoter-binding protein gene, a pentatricopeptide (PPR) repeat-containing protein gene, a BolA-like family protein gene, *AGO1* and a gene of unknown function. We also demonstrated that the DCL3-dependent rasiRNA biogenesis pathway, which had been considered absent in conifers, existed in Chinese fir. Furthermore, the miR390-TAS3-ARF regulatory pathway was elucidated.

**Conclusions:**

We unveiled a complex population of sRNAs in Chinese fir through high throughput sequencing. This provides an insight into the composition and function of sRNAs in Chinese fir and sheds new light on land plant sRNA evolution.

## Background

Identification and characterization of diverse classes of small non-coding RNAs (sRNAs) in eukaryotes has been a major research focus in recent years [1,2]. At least four classes of sRNAs have been well characterized in plants, including heterochromatic and repeat-associated small interfering RNAs (rasiRNAs) [3], microRNAs (miRNAs) [4,5], *trans*-acting siRNAs (tasiRNAs) [6] and natural antisense transcript-derived siRNAs (nat-siRNAs) [7]. In plants, the majority of endogenous sRNAs are 24-nt rasiRNAs [8]. They repress transposable elements and maintain genome integrity though guiding DNA methylation and histone modification [9-11]. To date, the DCL3-dependent rasiRNA biogenesis pathway has been reported in angiosperms and mosses [12], but considered absent from conifers [13]. MiRNAs are produced from primary miRNAs (pri-miRNAs) and precursor miRNAs (pre-miRNAs) by DICER LIKE 1 (DCL1) cleavage [14,15]. Mature miRNAs guide the RNA-induced silencing complexes (RISCs) to degrade target mRNA transcripts [16] or inhibit their translation [17]. MiRNAs play key roles in plant development, growth, nutrient homeostasis and responses to biotic and abiotic stresses [18]. In *Arabidopsis*, tasiRNA biogenesis initiates from miRNA-mediated cleavage of a non-coding primary transcript originated from the *TAS* locus [6,19]. RNA-dependent RNA polymerase 6 (RDR6) synthesizes long double-stranded RNA (dsRNA) molecules, which are sliced by DCL4 into phased 21-nt tasiRNAs [20]. In *Arabidopsis*, the miR390-TAS3-AUXIN RESPONSE FACTOR (ARF) pathway executes important functions in leaf development and lateral root formation [21]. Finally, nat-siRNAs are produced through natural antisense transcription and are fashioned by DCL1 or DCL2 [22]. The functional roles for eukaryotic nat-siRNAs that has been described to date are in environmental stress responses and developmental processes [23].

Identification of conserved and species-specific miRNAs usually relies on two approaches: computational prediction and experimental sequencing. By searching genomic and/or EST databases for orthologous sequences of known miRNAs and analyzing their pre-miRNA hairpin structures, many conserved miRNAs are identified from a variety of plants, such as *Arabidopsis thaliana*[24] and *Brassica napus*[25]. High throughput sequencing technologies, such as massively parallel signature sequencing (MPSS), 454 and sequencing-by-synthesis (SBS), have greatly facilitated the discovery of low abundant and recently evolved miRNAs in diverse plants, e.g., *Triticum aestivum*[26], *Oryza sativa*[27], and *Solanum lycopersicum*[28].

There have been reports of investigation and characterization of miRNAs in gymnosperms. Lu et al. identified 37 miRNAs from stem xylem of *Pinus taeda*, of which 6 miRNAs were likely associated with the fusiform rust gall disease [29]. By sequencing of small RNA libraries generated from a *Taxus chinensis* cell line, Qiu et al. found that the expression levels of 17 miRNAs have been significantly altered after treatment with methyl jasmonate [30]. Recently, a total of 18 conserved and 53 novel miRNA families were revealed in *Pinus contorta*[31]. Study on miRNAs in *Picea abies* indicated that 7 conserved and 9 novel miRNAs participated in the temperature-dependent epigenetic memory and climatic adaptation [32]. These reports showed that like in angiosperms, miRNA-guided post transcriptional gene regulation mechanism is important for the development, growth, stress responses and a myriad of other physiological processes in gymnosperms.

Chinese fir (*Cunninghamia lanceolata* Lamb. Hook) is one of the most important coniferous evergreen tree species in terms of both industrial and commercial wood supplies in China [33]. However, to date only 407 Chinese fir EST sequences are available in the public databases. No study on Chinese fir sRNAs has been reported so far. To gain mRNA transcriptome sequences of Chinese fir, we recently conducted a high-throughput sequencing of mRNAs isolated from a mixture of tissues. In this study, we used the sequencing-by-synthesis (SBS) technology to sequence a Chinese fir sRNA library and obtained a comprehensive set of sRNAs. Furthermore, we studied expression patterns of conserved and novel miRNAs by qRT-PCR. Potential targets were predicted for most miRNAs, of which 5 target genes have been experimentally verified. Intriguingly, the DCL3-dependent rasiRNA biogenesis pathway which had been thought to be absent in conifers was found in Chinese fir. These results suggest that regulative miRNAs exist in the economically important conifer, Chinese fir, and shed new light on the sRNA evolution from mosses to flowering plants.

## Results

### Chinese fir has a complex small RNA population

To identify sRNAs involved in Chinese fir development and growth, we used Illumina high throughput sequencing technology to sequence a sRNA library generated from total RNAs of seeds, seedlings, leaves, stems and calli. In total, 15,702,980 raw sRNA sequence reads were acquired and further processed with the BGI sRNA analysis pipeline. After removal of low-quality sequences, adapter sequences, polyA sequences, sequences smaller than 18 nt and other artifacts, we obtained 2,815,874 (13,246,904 raw reads, 84% of the total) unique sRNAs with lengths of 18 to 30 nt (Table 1).

**Table 1:** Statistics of sRNAs

**Category**	**Reads**	Percent (%)*
Total raw reads	15,702,980	
High-quality reads	14,682,846	93.5
3' adaptor null reads	4357	0.00003
Insert null reads	22,358	0.14
5' adaptor contaminant reads	115,878	0.74
Small insert reads (<18 nt)	1,290,435	8.22
Poly(A) sequence reads	2914	0.00002
Total clean sRNA reads	13,246,904	84.36
Unique sequence reads (18–30 nt)	2,815,874	17.93
Singleton sequence reads	2,075,775	13.22
Unique sequence reads (>2 reads)	740,099	4.71

^*^ The ratio is equal to the separate reads divided by the total raw reads.

We then mapped the 2,815,874 unique sRNA sequences to Chinese fir mRNA transcriptome sequences and *Arabidopsis thaliana* and *Populus trichocarpa* genome sequences, utilizing the computational software SOAP (http://soap.genomics.org.cn), owing to the lack of *C. lanceolata* genomic sequences. The Chinese fir mRNA transcriptome database contains 525,706 contig sequences, 84,980 scaffold sequences and 59,669 unigene sequences. The unigene sequences have an average length of 497 bp. The numbers of unique sRNAs perfectly matched the Chinese fir mRNA transcriptome sequences, the *A. thaliana* genome and the *P. trichocarpa* genome were 35,709 (991,997, 7.5%), 73,317 (6,612,119, 50%) and 66,269 (5,825,093, 44%), respectively (Table 2 and Table 3). A total of 7882 unique sRNAs were identified to be the common sRNAs matching perfectly to the Chinese fir mRNA transcriptome, *A. thaliana* genome and *P. trichocarpa* genome (Additional file 1). These perfectly aligned sRNAs are of particular interest as they represent highly conserved sRNAs in the three distantly related organisms. After further removal of rRNAs (2,929,163), tRNAs (1,611,677), snRNAs (6415), snoRNAs (2428) and repeat regions (736,626), a total of 8,697,221 sRNAs were obtained.

**Table 2:** **Annotations of sRNAs perfectly matching*A. thaliana*and*P. trichocarpa*genomes**

	***A. thaliana***		***P. trichocarpa***	
Class^*^	**Unique**	**Total**	**Unique**	**Total**
Match genome	73,317	6,612,119	66,269	5,825,093
Known miRNA	584	1,402,527	375	1,383,562
r/t/sn/snoRNA	39,304	5,136,628	35,706	3,097,919
Repeat	2603	10,280		
Exon-sense	21,764	35,844	6937	127,584
Exon-antisense	2171	6126	7125	608,685
Intron-sense	239	435	542	2221
Intron-antisense	244	681	678	1134
Un-annotated	6408	19,598	14,906	603,988

^*^sRNA categories, repeat, exon-sense, exon-antisense, intron-sense and intron-antisense, contain sRNAs perfectly matching the repeat sequences in the GenBank databases and Rfam database, the sense strand of a protein coding gene exon, the antisense strand of a protein coding gene exon, the sense strand of a protein coding gene intron and the antisense strand of a protein coding gene intron, respectively.

**Table 3:** Annotations of sRNAs perfectly matching Chinese fir mRNA transcriptome

**Class**	**Unique**	**Percent**	**Redundant**	**Percent**
Total	35,709	1.27%	991,997	7.49%
rRNA	90,192	3.20%	2,929,163	22.11%
tRNA	18,394	0.65%	1,611,677	12.17%
snRNA	2004	0.07%	6415	0.05%
snoRNA	894	0.03%	2428	0.02%
repeat	12,017	0.43%	736,626	5.56%
miRNA	31,979	1.14%	1,789,329	13.51%
Known miRNA (exact match)	115		1,301,346	9.82%
Novel miRNA	1		17	
Un-annotated	2,672,411	94.91%	6,907,892	52.15%
Total clean reads	2,815,874	100%	13,246,904	100%

Although some sRNAs were abundant and presented hundreds of thousands times in our database, the majority of sRNAs were sequenced only a few times. For example, 2,075,775 (74%) sRNAs were sequenced only once, which suggests that Chinese fir contains a large and complex sRNA population. The sRNA singleton rate of Chinese fir (74%) was similar to that of *A. thalianaO. sativa*, and *P. trichocarpa*, which is 65% [34], 82% [27], and 73% [35], respectively.

**Figure 1:**
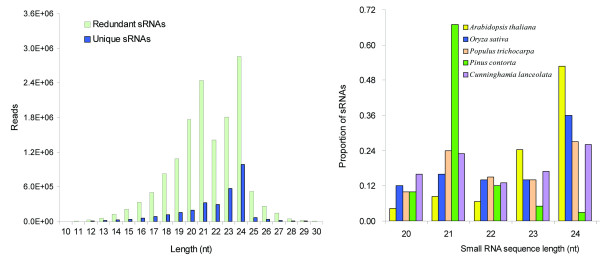
**Small RNA length distribution pattern.** (**A**) Length distribution and composition of Chinese fir unique sRNAs (dark blue bars, unique sRNAs; pale green bars, redundant sRNAs). (**B**) Comparison of sRNA length distribution pattern among 5 plant species.

The length distribution of unique sRNAs (10–30 nt) was summarized in Figure 1A. It was worthy to note that the length of the Chinese fir sRNAs was not evenly distributed. Conspicuously, 24 (22%) and 21 (18%) nt sRNAs were the two major size classes, which are the typical length of plant rasiRNAs and miRNAs, respectively. Meanwhile, the ratio of unique reads to total reads for 24- and 21-nt sRNAs was 0.35 and 0.13 respectively, which means that the 24-nt sRNAs have a higher complexity than the 21-nt sRNAs. The sRNA population yielded a median length of 21 nt and a low variance (σ = 7.5). We also compared the length distributions of sRNAs from 5 plant species, including *A. thaliana*, rice, *P. trichocarpa*, *P. contorta* and Chinese fir (Figure 1B). It showed that approximately 80% of the sRNAs ranged from 21 to 24 nt and the most abundant class of sRNAs was 21- or 24-nt sRNAs in these five species.

### Discovery of conserved miRNAs

To identify conserved miRNAs in Chinese fir, we conducted a local BLASTN search using unique sRNAs (2,815,874) against known plant miRNAs in the miRBase (version 17.0), which contains 3993 miRNAs from 47 plant species (13 April 2011). A total of 115 conserved miRNAs were identified in Chinese fir, belonging to 40 miRNA families (Additional file 2). Ninety-one (79%) conserved miRNAs start with a 5' terminal uridine residue, a conserved feature of miRNAs recognized by the AGO1 protein [26]. In addition, 584 unique sRNAs (1,402,527, 11%) perfectly matched *A. thaliana* miRNA precursors and 98 sRNAs were identical to *A. thaliana* mature miRNAs (232), whereas 375 sRNAs (1,383,562, 10%) mapped to *P. trichocarpa* miRNA precursors and 121 sRNAs exactly matched *P. trichocarpa* mature miRNAs (234) (Table 2). These figures suggest that approximately half of mature miRNAs are conserved between *C. lanceolata* and the two model angiosperms.

**Figure 2:**
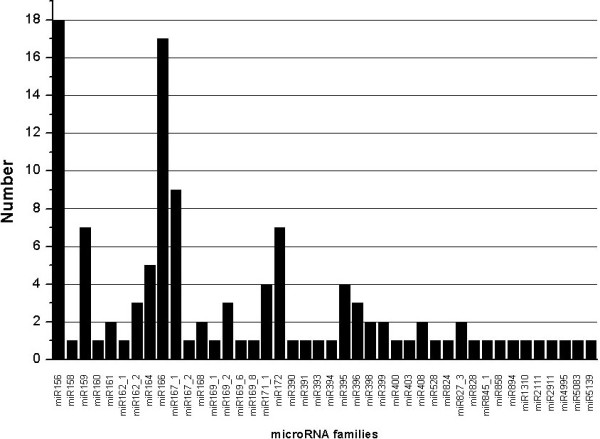
Numbers of miRNA member in each family in Chinese fir.

The high throughput sequencing technology provides an alternative way to assess expression profiles of diverse miRNA genes and the number of reads can serve as an index for the relative abundance of diverse miRNAs [31,36]. Intriguingly, Chinese fir miRNA abundance varied drastically. For example, cln-miR157a, cln-miR156a and cln-miR167a had 604,506, 514,552 and 47,800 redundancies, respectively, while many miRNAs (e.g., cln-miR156o, cln-miR159d and cln-miR319b) were sequenced only once. Similarly, the number of members within a miRNA family differed tremendously. For example, cln-MIR156 and cln-MIR166 families contained 18 and 17 members respectively, whereas many miRNA families (e.g. cln-MIR158, cln-MIR160 and cln-MIR390) possessed only one member. The detailed family member numbers were summarized in Figure 2. A total of 17 conserved miRNA families contained more than one member.

In the present study, we have tried to find the precursor sequences for the conserved Chinese fir miRNAs. Precursor sequences of 4 miRNAs, including cln-miR162d, cln-miR164b, cln-miR166o and cln-miR1310, have been identified from the Chinese fir mRNA transcriptome database (Additional file 3). Their hairpin structures predicted by MFOLD were shown in Additional file 4. The length of Chinese fir miRNA precursors ranged from 79 to 164 nt, with a majority of which (75%) ranging from 67 to 150 nt. This result is similar with that found in *Arabidopsis* and rice [37]. The minimal folding free energy indices (MFEIs) of Chinese fir miRNA precursors varied from 0.64 to 1.15, with an average of 0.83, in agreement with that of other plant miRNAs, such as *Arabidopsis*, rice, *Glycine maxMedicago truncatulaSaccharum officinarumSorghum bicolor* and *Zea mays*[38]. With complete genome sequences and larger EST databases, more precursor sequences will be found in Chinese fir. In addition, we identified the miRNA* sequences of cln-miR162d, cln-miR164b and cln-miR1310 in at least one of the three small RNA libraries constructed from total RNAs of Chinese fir cambium, providing further evidence that the three miRNAs are canonical miRNAs.

**Figure 3:**
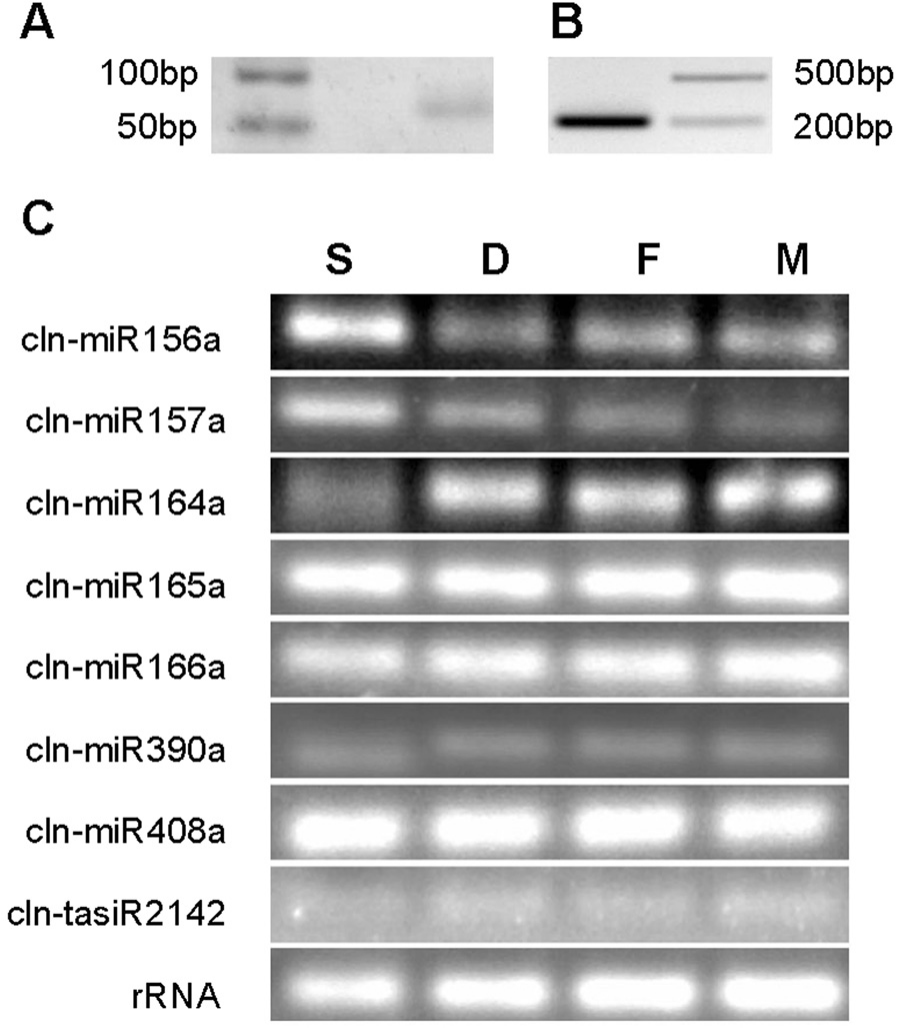
**RT-PCR and semi-quantitative RT-PCR analyses of conserved and novel microRNAs (miRNAs) and tasiRNA in Chinese fir.** (**A**) Validation of mature sequence of cln-miRn1. (**B**) Validation of precursor sequence of cln-miRn1. (**C**) Conserved miRNAs and tasiR2142. S, seeds; D, seedlings; F, leaves; M, stems.

We also performed RT-PCR analyses to confirm the expressions of 16 conserved mature miRNAs (cln-miR156a, cln-miR157a, cln-miR158a, cln-miR161a, cln-miR164a, cln-miR164b, cln-miR165a, cln-miR166a, cln-miR168a, cln-miR169a, cln-miR171a, cln-miR172a, cln-miR390a, cln-miR408a, cln-miR824 and cln-miR894) and cln-tasiR2142 (Additional file  5 and Figure 3). The results showed that these conserved miRNAs and cln-tasiR2142 could be detected in one or more of the four Chinese fir samples, including seeds, seedlings, leaves and stems. Further subcloning and sequencing of the PCR products confirmed that the mature sequences of 8 miRNAs (cln-miR157a, cln-miR164a, cln-miR165a, cln-miR166a, cln-miR171a, cln-miR390a, cln-miR408a and cln-miR894) and the precursor sequence of cln-miR164b were identical to the sequences obtained from Illumina sequencing.

After Blastp searches and further sequence analyses, a variety of unigene sequences in the Chinese fir mRNA transcriptome database were identified homologous to known proteins associated with miRNA biogenesis and action (Additional file 6). For example, 37 and 67 unigene sequences were found highly homologous to *Arabidopsis* DCL1 and AGO1 protein genes, respectively. Together with the finding of plenty of mature miRNAs and their precursors, these data provide clear evidence that the conserved pathway for miRNA generation and action exists in Chinese fir.

### Identification of novel miRNAs

The primary criterion for plant miRNA designation is the precise excision of an ~21-nucleotide miRNA/miRNA* duplex from the stem of a single stranded, stem-loop precursor [39]. Using the algorithm MIREAP, we found one putative novel miRNA, designated as cln-miRn1, in Chinese fir. The mature and precursor sequences of cln-miRn1 were further validated by subcloning (Figure 3A and B). The precursor sequence of cln-miRn1 can form canonical hairpin structure as predicted by MFOLD and has an MFEI value of 1.18, which is similar to that of other plant miRNAs (Additional file 2). The mature sequence of cln-miRn1 also begins with a 5' terminal uridine residue. The Illumina sequencing redundancy of cln-miRn1 is 17, which is much lower than that of many conserved miRNAs. The low redundancy of cln-miRn1 indicates that it may play important roles under certain conditions or in particular developmental stages in Chinese fir. Although the miR* sequence of cln-miRn1 was not identified in the sRNA database of the present study, we found it was in a small RNA library generated from total RNA of Chinese fir cambium. Using BLASTN, we did not find homologous sequence of unigene1397, the primary sequence of cln-miRn1, in the public databases. These results indicate that cln-miRn1 may be a novel and Chinese fir-specific miRNA.

### Expression profiles of conserved and novel miRNAs

To provide clues about the physiological functions of sRNAs in Chinese fir development and growth, we utilized semi-quantitative RT-PCR and real time RT-PCR to examine the expression profiles of 7 conserved miRNAs (cln-miR156a, cln-miR157a, cln-miR164a, cln-miR165a, cln-miR166a, cln-miR390 and cln-miR408a), the novel miRNA, cln-miRn1 and cln-tasiR2142 in samples of diverse developmental stages, including seeds, seedlings, leaves and stems. These sRNAs were selected for their key roles in plant development and growth [4,21,40-42].

**Figure 4:**
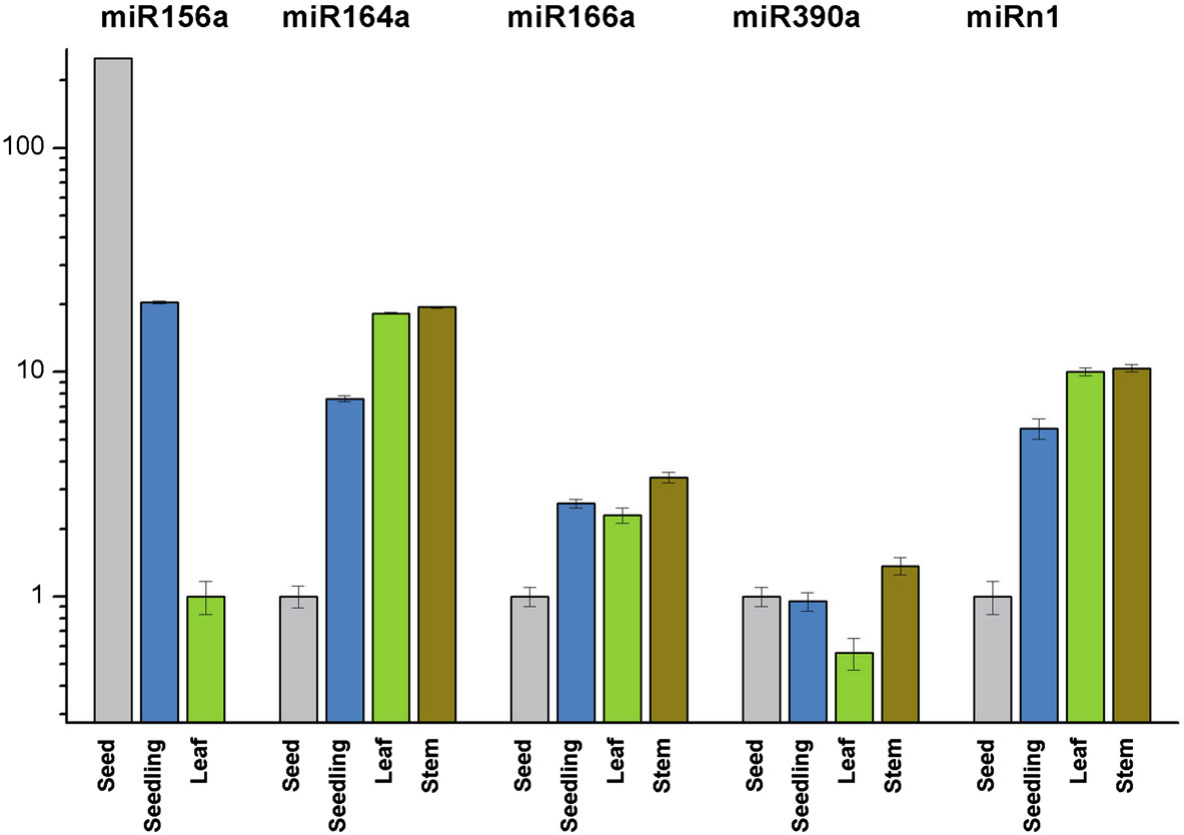
**Real-time RT-PCR analyses of conserved and novel miRNAs in Chinese fir.** Transcript levels were normalized to the arithmetic mean of the selected 5.8 s rRNA gene (for mature miRNAs) or *gapdh* (for miRNA precursor). Bars indicated the standard error of means.

The signal intensity on agarose gels demonstrated that cln-miR156a and cln-miR157a seemed to be strongly expressed in seeds but was barely detected in leaves and stems (Figure 3C). Consistent with this result, the expression level of cln-miR157a was more than 250-fold higher in seeds than in leaves (Figure 4). Conversely, cln-miR164a and cln-miRn1 were expressed abundantly in leaves and stems, moderately in seedlings and weakly in seeds. The expression levels of miRn1 were about 10-fold higher in leaves and stems than in seeds and twice higher than in seedlings. Cln-miR166a had moderate expressions in seedlings, leaves and stems and week expression in seeds. Cln-miR408a and cln-miR165a showed strong and ubiquitous expressions in the samples examined. These results indicate that the expressions of 7 conserved miRNAs, cln-miRn1 and cln-tasiR2142, as demonstrated by Illumina sequencing, were validated by RT-PCR or qRT-PCR. Some of them were expressed ubiquitously in all samples, while others displayed tissue and/or growth stage specific expression patterns.

### Prediction of miRNA targets

To better understand the functions of conserved and novel miRNAs, we predicted their putative targets by employing the Web-based psRNATarget program [43]. A total of 209 Chinese fir unigene sequences were predicted to be putative targets of 29 conserved miRNA families and one novel miRNA family (Table 4 and Additional file 7). Among them, 21 (10%) miRNA targets are homologous to the previously confirmed or predicted targets in *A*. *thalianaO. sativaP. trichocarpaP. taeda*, and/or *T. chinensis*. Four miRNA families (cln-MIR156, cln-MIR172, cln-MIR400 and cln-MIR858) have more than one conserved target, while 6 miRNA families (cln-MIR158, cln-MIR164, cln-MIR168, cln-MIR394, cln-MIR408 and cln-MIR828) have only one conserved target. These conserved targets include not only essential transcription factors, but also structural and metabolism-related proteins. For example, squamosa promoter-binding proteins (SBPs), important transcription factors known to control developmental timing, were predicted to be targets of cln-MIR156; the APETALA2-LIKE protein, an important transcription factor known to regulate flower development, was predicted to be a target of cln-MIR172 and non-transcriptional factor protein AGO1, an indispensable component for miRNA action, was predicted as a target of cln-MIR168.

**Table 4:** Conserved miRNA targets and their putative functions

**miRNA**	**Target function**	**Target**^**a**^	**Conserved with**^**b**^				
			**ath**	**osa**	**ptc**	**pta**	**tch**
Cln-miR156	SBP-domain protein	Unigene2030 (2)	+	+	+	+	+
		Unigene2872 (2)					
Cln-miR158	Unknown	Unigene28340 (4)	+	–	–	–	–
Cln-miR164	Pentatricopeptide repeat-containing protein	Unigene7992 (3)	+	+	+	–	+
Cln-miR168	Argonaute protein	Unigene6526 (3)	+	+	+	+	+
Cln-miR172	APETALA2-like protein	Unigene17425 (1.5)	+	+	+	+	+
		Unigene56981 (0.5)					
Cln-miR394	BolA-like family protein	Unigene56583 (4)	+	–	+	–	–
Cln-miR400	Pentatricopeptide repeat-containing protein	Unigene49313 (2)	+	–	–	–	–
		Unigene53642 (3)					
Cln-miR408	Basic blue copper protein	Unigene57297 (2)	+	+	–	–	–
Cln-miR828	MYB transcription factor	Unigene2867 (2)	+	–	–	–	–
		Unigene15489 (2.5)	+	–	–	–	–
		Unigene42567 (2.5)					
Cln-miR858	MYB transcription factor	Unigene58181 (2.5)					
		Unigene22248 (2)					
		Unigene6437 (3)					

^a^ All predicted miRNA targets with penalty scores (shown in parentheses) of 4 or less are listed.

^b^Ath, osa, ptc, pta and tch represent abbreviations for *A. thaliana*, *O. sativa*, *P. trichocarpa*, *P. taeda* and *T. chinensis*, respectively.

A total of 188 putative targets were not conserved in other plant species. Among them, 55 (26%) targets currently have no functional annotation. We were unable to predict targets for 10 conserved miRNA families. It may be attributable to insufficient Chinese fir mRNA sequences. Four unigene sequences were predicted to be targets of the novel miRNA, cln-miRn1. One of the 4 targets is similar to glycosyltransferase from *Lycium barbarum*, one is homologous to Cl-channel clc-7 from *P. trichocarpa*, whereas the other two have no functional annotation. This implies that cln-miRn1 may be involved in multiple physiological and metabolic processes in Chinese fir. More studies need to be performed to elucidate the functions of cln-miRn1 in the growth and development in Chinese fir.

### Validation of miRNA-guided cleavage of mRNAs

Plant mature miRNAs can guide RISC complexes to cleave target mRNAs through nucleotide complementarity. The cleavage site usually corresponds to the tenth nucleotide from the 5' end of the miRNA [44,45]. To verify that miRNAs can regulate their target mRNA expression in Chinese fir, we performed a modified RNA ligase-mediated rapid amplification of cDNA ends (RLM-RACE) experiment, using total RNA extracted from 2-month-old seedling stems (see Methods).

**Figure 5:**
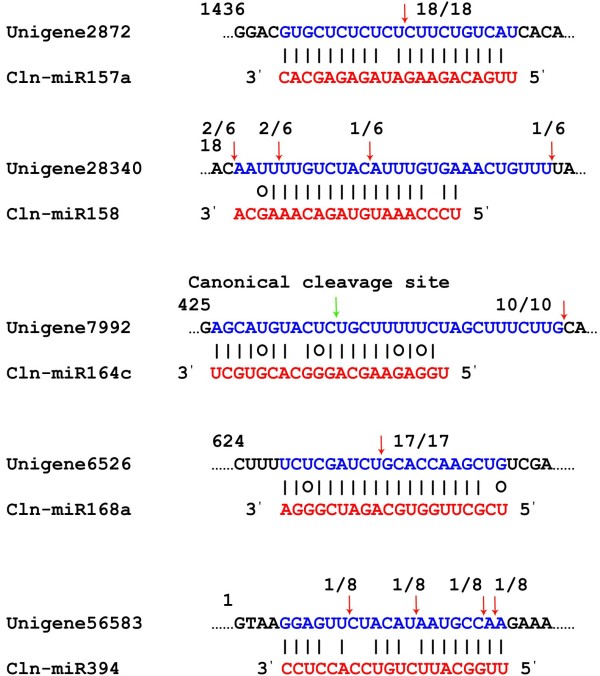
**Detection of miRNA-mediated mRNA cleavage using modified 5' RACE.** Partial mRNA sequences from target genes were aligned with corresponding miRNAs. Each top strand (blue) represents a miRNA complementary site in the target mRNA, and each bottom strand (red) represents the miRNA. G:U wobble pairing (circles) and Watson-Crick pairing (vertical dashes) are indicated. Red arrows indicate the 5' termini of the degraded mRNA fragments isolated from Chinese fir, which were identified from cloned 5' RACE products, with the frequency of clones shown.

In the present experiment, 5 unigene sequences were verified to be the targets of 5 Chinese fir miRNAs. Sequencing of the 5' RACE cleaved product of unigene2872 identified a precise slice at the cln-miR157a binding site, between position 10 and 11 (Figure 5). Unigene2872 encodes a protein homologous to the *Arabidopsis* transcription factor squamosa promoter-binding protein-like 7 (SPL7). Unigene6526 encoding AGO1 protein had a main cleavage site at the 11th nucleotide of cln-miR168a from the 5'-end. Unigene56583 and unigene28340 were validated to be targets of cln-miR394 and cln-miR158, respectively, with multiple cleavage sites. Unigene56583 codes for a protein highly homologous to BolA-like family protein, while unigene28340 has no annotation in the public databases. Unigene7992, the putative target of cln-miR164c, was also evaluated for its cleavage site. It was sliced 21 nucleotides downstream the canonical cleavage site, which could be attributed to secondary siRNA in the 21-nucleotide register with the cleavage site for miRNAs, as reported by Ronemus and De Paola [46,47]. Unigene7992 codes for a protein highly homologous to pentatricopeptide (PPR) repeat-containing protein.

### Identification of DCL enzymes and phylogenetic analysis

**Figure 6:**
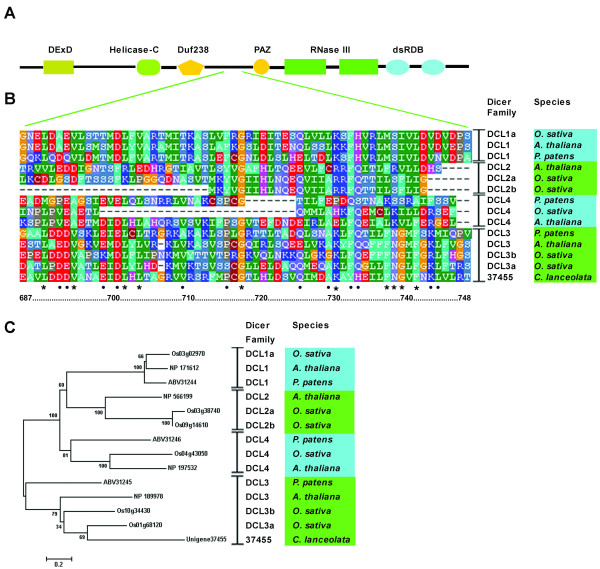
**Dicer alignment and phylogenetic analysis.** (**A**) Domain organization of an angiosperm Dicer protein. (**B**) Amino acid sequences corresponding to unigene37455 from Chinese fir and the regions between Duf238 and PAZ domain from 4 *A. thaliana*, 6 *O. sativa* and 3 *P. patens* Dicers were aligned using Bioedit. Only the alignment of the region exhibiting the highest sequence conservation was shown (62 amino acids). Ten residues were absolutely conserved in this region and 12 residues were highly conserved, marked with asterisks and dots, respectively. (**C**) Phylogenetic tree of Dicers generated from the alignment of unigene37455 and three model plant Dicer proteins.

Because DCL enzymes are crucial determinants of small RNA size, we proceeded to identify candidate DCL proteins in Chinese fir. Figure 6A shows the linear organization of an angiosperm Dicer protein that contains DexD-helicase, helicase-C, Duf283, PAZ, RNase III and double-stranded RNA-binding (dsRBD) domains. However, DCL2 lacks a second dsRBD and DCL3 lacks Duf283. Given that Chinese fir genome has not been sequenced, we searched Chinese fir mRNA transcriptome database for putative DCLs using 4 known Dicer nucleotide sequences from *A. thaliana*. After Blastp searches and further sequence analysis, 29 unigene sequences were identified orthologous to *Arabidopsis* DCL proteins with high-quality alignments (>35% similarity on the amino acid level, Additional file 8). The predicted amino acid sequences of 18 unigenes were found containing diverse DCL domains. Five of the 6 known dicer domains were identified (DexD, helicase-C, PAZ, RNase III and dsRBD), whereas the Duf283 domain was not found. Multiple sequence alignments were made with predicted DCL sequences and the corresponding regions from each of the 4 *A. thaliana*, 6 *O. sativa* and 3 *P. patens* DCLs. Figure 6B shows the most conserved regions (62 amino acids long) of unigene37455 and the three model plant DCLs. Ten residues were absolutely conserved in this region and 12 residues were highly conserved (Figure 6B, marked with asterisks and dots, respectively). The alignments were used to generate the most likely phylogenetic tree of Dicers (based on 1000 rounds of bootstrapping, Figure 6C, Additional file 9). Three unigene sequences (unigene18579, unigene 37455 and unigene16720) aligned very well with angiosperm and fern DCL1, DCL3 and DCL4, respectively. Together with the identification of highly conserved rasiRNAs, miRNAs and tasiRNAs in Chinese fir, these results provide compelling evidence for the conservation of sRNA-generating pathways between gymnosperms and angiosperms.

### DCL3-dependent rasiRNA generation pathway exists in Chinese fir

It has been well-characterized that DCL3 pays key roles in the biogenesis of 24-nt rasiRNAs, the major endogenous sRNAs in both *Arabidopsis*[48] and moss *Physcomitrella patens*[12]. However, reports on small RNAs from gymnosperms, e.g., *T. chinensis*[30] and *P. contorta*[31], showed that they synthesize a diverse population of small RNAs that are 21-nt long, while fail to produce significant amounts of 24-nt sRNAs. Consistently, DCL3, the enzyme responsible for the maturation of 24-nt rasiRNAs in angiosperms, appeared to be absent from conifers [13]. In this study, we found that the length distribution of Chinese fir sRNAs was similar to that of many angiosperms (e.g., *A. thaliana* and trifoliate orange) in having a major peak at 24 nt. In addition, 3 unigene sequences (unigene34518, unigene35698 and unigene37455) were identified highly homologous to *O. sativaP. trichocarpa* and *P. patens* DCL3 proteins. Moreover, we revealed other unigene sequences homologous to known proteins necessary for biogenesis and action of rasiRNAs from the Chinese fir mRNA transcriptome database, including RNA polymerase IV (21 unigene sequences), RDR2 (10 unigene sequences), HEN1 (1 unigene sequence), AGO4 (7 unigene sequences) and AGO6 (4 unigene sequences) (Additional file 10). Besides,12,017 unique sRNAs (736,626) were able to map to the sense or antisense strand of known repeat-associated RNA sequences using BLASTN analysis against the Repeat database (http://www.repeatmasker.org/cgi-bin/WEBRepeatMasker) (Table 3), indicating that these sRNAs may originate from pericentromeric regions, transposons or repetitive elements. Taken together, these results suggest that DCL3-dependent rasiRNA generation pathway may exist in Chinese fir, a gymnosperm species.

### MiR390-TAS3-ARF pathway

TasiRNAs belong to a plant-specific class of endogenous sRNAs. Previous studies showed that one of the conserved tasiRNAs, TAS3, plays key roles in mosses and flowering plants [19]. However, TAS3-related genes have not been reported in gymnosperms so far. In the present investigation, we found not only one MIR390 family member, but also three small RNA sequences identical to *Arabidopsis TAS3* siRNAs, including siR1778, siR1769 and tasiR2142, in the Chinese fir sRNA library (Additional file 2). To confirm the expression of cln-miR390a and cln-tasiR2142 in Chinese fir, we performed relative real-time and semi-quantitative RT-PCR analyses. The transcription levels of cln-tasiR2142 were low and did not show significant differences in the four samples examined (Figure 3). Cln-miR390a was weakly expressed in seeds, seedling and stems and very weakly in leaves (Figures 3 and 4). In addition, we identified unigene sequences homologous to known proteins necessary for tasiRNA biogenesis and action, e.g., RDR6 (11 unigene sequences), DCL4 (29 unigene sequences) and AGO7 (15 unigene sequences) from our mRNA transcriptome database (Additional file 11). Using the Web-based target prediction program psRNATarget and Chinese fir mRNA transcriptome database and 61,864 contigs from *P. taeda* (DFCI Pine Gene Index release 7.0) as the target databases, we found 2 contigs (TC109284 and TC85135) annotated as *ARF*s in *P. taeda* to be targets of cln-tasiR2142. Thus, our study provides strong evidence that this crucial and conserved miR390-TAS3-ARF regulatory pathway may exist and play important roles in Chinese fir.

## Discussion

### sRNA length distribution divergence among gymnosperms

Previous studies showed the sRNA length distribution patterns of many angiosperms, e.g., *Arabidopsis thaliana*[34], *Citrus trifoliata*[49] and *Medicago truncatula*[50], and mosses [12] have a major peak at 24 nt. However, the length distribution patterns of sRNAs from two gymnosperms, *Taxus chinensis*[30] and *Pinus contorta*[31], constitute a major 21-nt peak and no obvious 24-nt peak is observed. A survey of sRNAs in 24 vascular plant species revealed that 7 conifer species fail to produce significant amounts of 24-nt sRNAs [13]. Therefore, it seems that the sRNA length distribution patterns are significantly divergent between angiosperms and gymnosperms.

In the present study, we found that Chinese fir sRNA length distribution pattern was similar to that of many angiosperms, having a 24-nt major peak. We speculate that the divergence in sRNA length distribution pattern between Chinese fir and other gymnosperms may be due to two reasons. One reason is the cell or tissue type difference of the initial sample used to generate the small RNA library. Multiple tissues and organs in different developmental stages, including seeds, seedlings, leaves, stems and calli, were used as the initial sample in Chinese fir small RNA library construction, whereas only one tissue, a stem cell line of *T. chinensis*[30] or young needles of *Pinus contorta*[31] or young needle tips or leaf buds of 10 gymnosperms [13], were utilized as the initial samples in their experiments. Young needles, leaf buds or stems mainly consist of terminally differentiated cells, while our sample contains not only terminally differentiated cells, but also differentiating cells, such as cells in seeds and seedlings. Therefore, the divergence of sRNA length distribution patterns between Chinese fir and other gymnosperms may be partially caused by the sRNA population difference between differentiating and differentiated cells. The other reason that cannot be excluded is the species differences between Chinese fir and other gymnosperms.

### Conserved miRNAs in Chinese fir

In comparison with thousands of miRNAs identified from angiosperms, the reported miRNAs from gymnosperms are limited. Only miRNAs of two conifer species, *Pinus taeda*[29] and *Picea abies*[32], are listed in the miRBase (version 17.0) with a total of 40 and 37 miRNAs respectively. No research has been conducted to identify miRNAs from Chinese fir, an economically important gymnosperm in China. In this report, we conducted the first screen for Chinese fir miRNAs by deep sequencing. A total of 115 conserved miRNAs belonging to 40 miRNA families were identified in Chinese fir. For the 21 common conserved miRNA families among three model angiosperms, *A. thalianaO. sativa* and *P. trichocarpa*, 20 miRNA families were found in Chinese fir, except the MIR397 family. These results imply that the ancient miRNA biogenesis and action system is well developed in the common ancestors of gymnosperms and angiosperms.

It is well-characterized that miR156 regulates the timing of the juvenile-to-adult transition by repressing the expression of *SQUAMOSA PROMOTER BINDING PROTEIN LIKE* (*SPL*) family of transcription factor genes in *Arabidopsis*[51]. Ath-miR156 is also involved in regulating the onset of flowering [52], temporal control of trichome distribution [53] and securing male fertility in *Arabidopsis*[54]. In the present study, we identified 18 miRNA members of the cln-MIR156/157 family by Illumina sequencing, of which cln-miR157a is the most abundant miRNA in the sRNA library. Using semi-quantitative and quantitative RT-PCR, we found that the transcript levels of cln-miR156a and cln-miR157a were down-regulated along the juvenile-to-adult transition (Figure 3 and Figure 4). Moreover, we predicted 40 mRNAs to be targets of cln-MIR156 family miRNAs (Table 4 and Additional file 7). The analysis of cln-miR156 targets showed that 2 targets were *SPL* family of transcription factor genes, including unigene2030 and unigene2872. In addition, unigene2872, encoding a protein homologous to the *Arabidopsis* transcription factor *SPL7*, was experimentally verified to be the target of cln-miR157a by 5' RACE. These results suggest that cln-miR156a and cln-miR157a may regulate juvenile-to-adult transition by down-regulating the expression of *SPL* transcription factor genes in Chinese fir.

In rice, *PETER PAN SYNDROME* (*PPS*), an *Arabidopsis CONSTITUTIVE PHOTOMORPHOGENIC1* (*COP1*) ortholog, regulates the juvenile-to-adult and vegetative-to-reproductive phase changes by controlling the expression of osa-miR156 [55]. In the present study, unigene9100, an *Arabidopsis COP1* ortholog, was predicted to be the target of cln-miR156, indicating that *COP1*-miR156 pathway may be self-regulated during the phase change in Chinese fir development. Furthermore, mRNAs involving in defense responses (e.g., unigene57989) and other metabolic pathways (e.g., unigene8339) were also predicted to be targets of cln-miR156. These results indicate that cln-miR156/157 family miRNAs may be involved in multiple physiological processes in Chinese fir development.

The functions of miR168 have been intensively studied in previous research. Várallyay et al. reported that plant viruses repress AGO1 accumulation by modulating endogenous miR168 level to alleviate the anti-viral function of AGO1 protein [56]. Recently, Li et al. revealed a transcriptional regulatory mechanism by which miR168 controls AGO1 homeostasis during ABA treatment and abiotic stress responses in *Arabidopsis thaliana*[57]. In the present study, we found not only 2 MIR168 family members, but also unigene6526, an ortholog of *Arabidopsis* AGO1, to be the target of cln-miR168a using 5' RACE. These results imply that cln-miR168a may have important roles in viral infection, stress responses and other physiological processes by repressing the expression of AGO1 in Chinese fir.

Previous studies have established that miR164 targets NAC family transcription factor is well conserved in many plant species and is involved in the trifurcate feed-forward pathway to ensure aging-induced leaf senescence [40] and in the lateral root development in *A. thaliana*[58]. In the present experiment, we identified 5 MIR164 family members and detected developmental stage-associated expression pattern of cln-miR164a in four Chinese fir samples by qRT-PCR. Moreover, unigene7992, a NAC family transcription factor, was verified to be the target of cln-miR164a. These results provide direct evidence that cln-miR164a may participate in multiple physiological processes during development and growth by inhibiting the expression of NAC transcription factors in Chinese fir.

### DCL3-dependent rasiRNA biogenesis pathway

The DCL3-dependent rasiRNA generation pathway has been reported in both angiosperms and mosses [8,12]. However, this pathway has been considered absent from gymnosperms because of the lack of significant amounts of 24-nt sRNAs and DCL3 in conifers [13].

The experiments described here demonstrated that 24-nt sRNAs were the most abundant size class of sRNAs in Chinese fir. In addition, we found that 736,626 sRNAs could map to the sense or antisense strand of known repeat-associated DNA sequences, suggesting that these sRNAs may be transcribed from pericentromeric regions, transposons or repetitive elements. Moreover, unigene sequences homologous to the proteins necessary for the biogenesis and action of the 24-nt rasiRNAs were identified from the Chinese fir mRNA transcriptome database, including *Pol IV*, *RDR2*, *DCL3*, *HEN1*, *AGO4* and *AGO6* (Figure 6 and Additional file 10). These results indicate that the ancient but crucial DCL3-dependent rasiRNA generation pathway may exist in Chinese fir, a gymnosperm species.

In rice, two DCL3 family members have distinct roles in the generation of 24-nucleotide small RNAs [59]. OsDCL3a plays conserved roles in producing 24-nucleotide unphased small RNAs, which can direct cytosine DNA methylation both in *cis* and in *trans*. OsDCL3b specifically acts in 24-nucleotide phased small RNA biogenesis, which exhibits panicle- and early seed-specific expression and plays roles in stamens. In *Physcomitrella patens*, DCL3 is required for the accumulation of 22–24 nt siRNAs, but not 21 nt siRNAs, at Pp23SR loci [13]. In the present investigation, we identified 3 unigene sequences, including unigene34518, unigene35698 and unigene37455, highly homologous to rice DCL3a, *Physcomitrella patens* DCL3, and *Populus trichocarpa* DCL3, respectively, suggesting that unigene34518 may be responsible for the biogenesis of 24-nucleotide unphased small RNAs, whereas unigene35698 and unigene37455 may be involved in the producing other classes of 24-nucleotide small RNAs in Chinese fir.

## Conclusions

Using deep-sequencing technology, we obtained a comprehensive set of sRNAs in Chinese fir, including rasiRNAs, conserved and novel miRNAs and tasiRNAs. A total of 115 conserved miRNAs comprising 40 miRNA families and one novel miRNA were identified. The expressions of 16 conserved and one novel miRNAs and one tasiRNA were examined by RT-PCR. Real-time quantitative PCR results demonstrated that four conserved and one novel miRNAs displayed developmental stage-specific expression patterns. In addition, 209 unigene sequences were predicted to be the targets of 30 Chinese fir miRNA families, of which five unigene sequences were experimentally verified by 5' RACE. The DCL3-dependent rasiRNA generation pathway, which had been considered absent in conifers, was found in Chinese fir. Furthermore, the miR390-TAS3-ARF regulatory pathway was investigated. In conclusion, global identification and characterization of sRNAs from Chinese fir expand our knowledge of conifer sRNAs and provide an insight into land plant sRNA evolution.

## Methods

### Plant material

Chinese fir plants were grown under standard greenhouse conditions. Seedlings, adult leaves and stems were harvested and stored at −80°C until use. Dry seeds were stored at 4°C. Calli derived from immature Chinese fir seeds were maintained on a Chinese fir-specific culture medium.

### Small RNA isolation and Illumina sequencing

Total RNAs of seeds and calli were extracted using RNAiso-mate for plant tissue and RNAiso plus (Takara, Dalian, Liaoning, China), whereas total RNAs of seedlings, adult leaves and stems were isolated with the Concert Plant RNA Reagent (Invitrogen, Carlsbad, CA, USA), and were then treated with RNase-free DNase I (Promega, Madison, WI, USA). Equal amount of total RNAs from the 5 different samples were mixed to form a single RNA pool. Twenty micrograms of total RNAs from the pool were used and 16 to 30-nt sRNAs were purified using Novex 15% TBE-Urea gel (Invitrogen). Two adaptors were sequentially ligated to the 5' and 3' ends of purified sRNAs. The ligation products were further purified from Novex 10% TBE-Urea gel. Reverse transcriptase SuperScript II (Invitrogen) and high-fidelity DNA polymerase Phusion (New England Biolabs, Ipswich, MA, USA) were used in the following RT-PCR reaction. The amplification products were cut from Novex 6% TBE-Urea gel. The purified DNA fragments were used for sequencing on an Illumina 1 G Genome Analyzer at the Beijing Genomics Institute, Shenzhen, China.

### SRNA sequence processing

The raw data were processed with the BGI sRNA analysis pipeline to filter out artifact sequences. Non-redundant sRNAs ranging from 18 to 30 nt were collected and stored in the Clean file and reads of unique sequences were recorded and submitted to NCBI [GEO: GSE24226]. Unique sRNAs in the Clean file were mapped to Chinese fir mRNA transcriptome database [GenBank:SRA053525] (Additional file 12) and *A. thaliana* and *P. trichocarpa* genomes using SOAP, according to its default settings (http://www.soap.genomics.org.cn). The *A. thaliana* genome sequences and their annotations were downloaded from the TIGR website (ftp://ftp.tigr.org/); The *P. trichocarpa* genome sequences and their annotations were downloaded from the JGI website (http://genome.jgi-psf.org/Poptr1_1/Poptr1_1.download.ftp.html). rRNA, tRNA, snoRNA and snRNA sequences were downloaded from NCBI and Rfam 9.0 (http://rfam.sanger.ac.uk/), and coordinates of genomic repeats were obtained from RepeatMasker (http://www.repeatmasker.org/PreMaskedGenomes.html). The perfectly aligned sRNA was annotated as rRNA/tRNA/snRNA/snoRNA, miRNA, repeat element, exon-sense, exon-antisense, intron-sense or intron-antisense and stored in separate files, based on the annotation of the sequence it overlapped in Chinese fir transcriptome, *A. thaliana* or *P. trichocarpa* genome. Matching sRNAs without annotation were stored in the Un-annotated file.

### Identification of conserved and novel miRNAs

To identify conserved miRNAs, unique sRNAs (2,815,874) from the sRNA library and contigs (525,706) from the Chinese fir mRNA transcriptome database were utilized in local BLASTN analyses (E value was set to 0.01 and mismatches were set to less than 3) against the mature and precursor sequences of miRNAs in miRBase version 17.0 (http://www.mirbase.org/) [60]. Overlapping contig sequences were used to form longer sequences according to their alignments to known miRNA precursor sequences in the miRBase. Sequences homologous to known miRNA precursors were annotated as candidate Chinese fir miRNA precursors. To further reveal conserved and novel miRNA precursors, the unique sRNAs (2,815,874) were aligned to the Chinese fir mRNA transcriptome database using MIREAP with default parameters (http://sourceforge.net/projects/mireap/). BLASTN searches against all nucleotide sequences in NCBI databases were performed to investigate whether these potential miRNA precursors were conserved in other plant species. Putative precursors homologous to known plant rRNAs, tRNAs or mRNAs were excluded. Mfold was used to predict the secondary structures of the candidate miRNA precursor sequences, utilizing default parameters (http://mfold.bioinfo.rpi.edu/cgi-bin/rna-form1.cgi) [61]. The minimal folding free energy (MFE) of the sequence was set to less than or equal to −30 kcal/mol. MiRNA precursors with minimal folding free energy index (MFEI) values less than 0.60 were also discarded [38]. Only the perfectly matched sRNA sequences and homologous sequences with precursor sequences were considered to be conserved miRNAs. Sequence with proper secondary hairpin structure, a MFEI value more than 0.60 and no homologous sequence in public databases was considered as putative novel miRNA precursor sequence.

### Semi-quantitative and real-time quantitative RT-PCR

Total RNAs were extracted separately from seeds, 2-month-old seedlings, adult leaves and stems as described above. For mature miRNA expression analysis, cDNAs were synthesized from 1 μg of purified total RNAs with the NCode miRNA First-Strand cDNA Synthesis Kit (MIRC-50; Invitrogen). Forward primers were designed based on mature miRNA sequences (Additional file 13). If the T_m_ of a mature miRNA was <60°C, it was adjusted by adding Gs or Cs to the 5' end and/or As to the 3' end of the miRNA sequence [32]. A primer corresponding to a 20-bp segment at the 3' end of a Chinese fir 5.8 s rRNA gene was used as a reference control. We adopted stringent annealing conditions and set the annealing temperature to 65°C for quantitative real-time RT-PCR reactions because many miRNA paralogs differ by only one nucleotide [62]. Relative real-time quantitative PCR was performed using Toyobo’s Thunderbird SYBR qPCR mix in a 20-μl reaction volume, using 2 μl of 1:10 diluted cDNA solution as template and 0.3 μM of each primer. Triplicate reactions were conducted on a quantitative PCR machine (MX3000P, Stratagene, La Jolla, CA, USA), using the following thermal cycling conditions: 95°C for 1 min, 45 cycles of 95°C for 15 s, 65°C for 15 s, and 72°C for 5 s.

For miRNA precursor expression analysis, cDNAs were synthesized from 2 μg of purified total RNAs in 25-μl reactions containing 200 U M-MLV reverse transcriptase (Promega) and 1 μg random nonamers, according to the manufacturer’s protocol. Six pairs of primers for 5 housekeeping genes, including actin, ubiquitin, *gapdh*, tubulin and translation factor EF-1 alpha-like gene and two pairs of primers for cln-miR164b and cln-miRn1 precursors were designed. Relative real-time quantitative PCR was carried out as described above using the following thermal cycling conditions: 95°C for 1 min, 40 cycles of 95°C for 15 s, 55°C for 15 s and 72°C for 10 s. Among the 5 housekeeping genes, *gapdh* had the most similar expression profiles in the four samples and was used as a reference gene.

After PCR, a thermal denaturing cycle was carried out to determine the dissociation curve and verify the specificity of the amplification. The amplification results were analyzed using a comparative C_t_ method, which used an arithmetic formula, 2^-ΔΔCt^. C_t_ represents the threshold cycle. All expression levels were normalized to the arithmetic mean of the selected 5.8 s rRNA gene (for mature miRNAs) or *gapdh* (for precursor). The expression level in seeds or leaves was arbitrarily set to 1.

Semi-quantitative RT-PCR was conducted with the following thermal cycling parameters: 95°C for 1 min, 95°C for 15 s, 65°C for 15 s, and 72°C for 5 s for 40 cycles. The final concentration of primers was 0.3 μM. We designed 17 forward primers for 16 conserved miRNAs and tasiR2142. Amplification products were separated with 2.5% agarose gel electrophoresis. Three RT-PCR replications for each primer were conducted with independently isolated total RNAs.

To further confirm the specificity of the amplification, we analyzed PCR samples on 2.5% agarose gels with EtBr visualization of bands. Fragments were gel-purified, cloned into the pGEM-T Easy vector (Promega) and sequenced.

### Target mRNA prediction

We used the Web-based psRNATarget program to identify putative targets for conserved and novel miRNAs (http://bioinfo3.noble.org/psRNATarget/). The custom plant transcript databases include 59,669 unigene sequences from the Chinese fir mRNA transcriptome database and 61,864 contigs from *P. taeda* (DFCI Pine Gene Index release 7.0, http://compbio.dfci.harvard.edu/cgi-bin/tgi/gimain.pl?gudb=pine). A scoring system was applied according to Zhang [43]. Sequences with a penalizing score ≤4 were chosen as putative targets.

### MiRNA-mediated cleavage of mRNA

For identification of internal cleavage sites in the target mRNAs, a modified RNA ligase-mediated rapid amplification of cDNA ends (RLM-RACE) experiment was carried out using a 5' RACE kit (Takara) [49,63]. Total RNA was extracted from 2-month-old seedling stems and purified as described above. An RNA Oligo adapter was directly ligated to the purified RNAs (2000 ng) without calf intestinal phosphatase and tobacco acid pyrophosphatase treatment. Thirty nesting and nested gene-specific primers were synthesized and used for PCR amplifications. Ten DNA bands with expected sizes were gel purified and cloned into the pGEM-T Easy vector and sequenced. Five of the sequenced DNA bands were found to be miRNA-guided cleavage products.

### Dicer alignment and phylogenetic analysis

DCL sequences from *A. thaliana* and *P. patens* were obtained from GenBank (http://www.ncbi.nlm.nih.gov/Genbank/index.html). *O. sativa* DCL sequences were accessed via the Institute for Genomic Research (TIGR) rice database (http://www.tigr.org/tigr-scripts/osa1_web/gbrowse/rice). Accession numbers are as follows: *A. thaliana*: NP_171612, NP_566199, NP_189978, NP_197532/DQ118423; *O. sativa*: Os03g02970, Os03g38740, Os09g14610, Os01g68120, Os10g34430, Os04g43050; *P. patens*: ABV31244, ABV31245, ABV31246.

Protein domains of the Chinese fir DCLs were analyzed by scanning them against the InterPro protein signature database (http://www.ebi.ac.uk/InterProScan). Domains were assigned according to pFAM predictions.

Dicer protein alignments were conducted using Bioedit with default parameters. The MEGA program was used to generate the phylogenetic tree of Dicers in *A. thaliana*, *O. sativa*, *P. patens* and Chinese fir, using the neighbor-joining algorithm with 1000 rounds of bootstrapping.

## Abbreviations

AGO, Argonaute; DCL, Dicer-like; EtBr, Ethidium bromide; HEN1, Hua Enhancer 1; MFEI, Minimal folding free energy index; miRNA, microRNA; pre-miRNA, precursor miRNA; pri-miRNA, Primary miRNA; RACE, Rapid amplification of cDNA ends; rasiRNA, repeat-associated small interfering RNA; RDR, RNA-dependent RNA polymerase; RISC, RNA-induced silencing complex; RLM-RACE, RNA ligase-mediated rapid amplification of cDNA ends; rRNA, ribosomal RNA; RT, Reverse transcription; snRNA, small nuclear RNA; snoRNA, small nucleolar RNA; tasiRNA, *trans*-acting small interfering RNA; tRNA, transfer RNA.

## Authors' contributions

LW and FW designed and carried out the study and drafted the manuscript. XG participated in the bioinformatics analysis. ZQ and YZ conducted the sequence alignment. HZ, SL and JL conceived of the study, participated in its design and coordination and helped to draft the manuscript. All authors read and approved the final manuscript.

## Supplementary Material

Additional file 1 **Common sRNAs matching perfectly to the Chinese fir mRNA transcriptome,*A. thaliana*and*P. trichocarpa*genomes.** Click here for file

Additional file 2 Conserved and novel miRNAs and tasiRNAs in Chinese fir. Click here for file

Additional file 3 Precursor and primary sequences of conserved and novel miRNAs in Chinese fir. Click here for file

Additional file 4 The hairpin structures of conserved and novel miRNAs predicted by MFOLD. Click here for file

Additional file 5 RT-PCR of conserved mature miRNAs. Click here for file

Additional file 6 Unigenes involved in the biogenesis and action of miRNAs in Chinese fir. Click here for file

Additional file 7 Conserved and novel miRNA targets and their putative functions. Click here for file

Additional file 8 Dicer-like proteins in Chinese fir. Click here for file

Additional file 9 Phylogenetic trees of unigene18579 and unigene16720 with three model plant DCLs. Click here for file

Additional file 10 Unigenes involved in the biogenesis and action of 24-nt rasiRNAs in Chinese fir. Click here for file

Additional file 11 Unigenes involved in the biogenesis and action of tasiRNAs in Chinese fir. Click here for file

Additional file 12 Total unigenes in Chinese fir. Click here for file

Additional file 13 Primers for RT-PCR, qRT-PCR and 5' RACE. Click here for file
