# Supplementary material for: Identification and characterization of small non-coding RNAs from Chinese fir by high throughput sequencing
Source: BMC Plant Biol. 2012 Aug 15;12:146. doi: 10.1186/1471-2229-12-146 (PMC3462689; doi:10.1186/1471-2229-12-146)
Supplement: Additional file 2 — Conserved and novel miRNAs and tasiRNAs in Chinese fir. [file 1471-2229-12-146-S2.doc]

**Additional file 2** Conserved and novel miRNAs and tasiRNAs in Chinese fir.

| **miRNA family** | **Name** | **Sequence (5'-3')** | **Length (nt)** | **Reference miRNA** | **Reads** | **RT-PCR** | **MFEI** |
| --- | --- | --- | --- | --- | --- | --- | --- |
| 156/157 | cln-miR156a | UGACAGAAGAGAGUGAGCAC | 20 | ath-miR156a | 514552 | + |  |
|  | cln-miR156b | UGACAGAAGAGAGUGAGCACA | 21 | bna-miR156a | 6020 |  |  |
|  | cln-miR156c | UGACAGAAGAGAGAGAGCAC | 20 | ahy-miR156a | 1260 |  |  |
|  | cln-miR156d | UGACAGAAGAGAGGGAGCAC | 20 | ptc-miR156k | 903 |  |  |
|  | cln-miR156e | UUGACAGAAGAGAGAGAGCAC | 21 | ahy-miR156c | 840 |  |  |
|  | cln-miR156f | UGACAGAAGAGAGCGAGCAC | 20 | zma-miR156k | 814 |  |  |
|  | cln-miR156g | UUGACAGAAGAAAGAGAGCAC | 21 | smo-miR156c | 563 |  |  |
|  | cln-miR156h | CGACAGAAGAGAGUGAGCAC | 20 | ath-miR156g | 535 |  |  |
|  | cln-miR156i | UUGACAGAAGAUAGAGGGCAC | 21 | mtr-miR156g | 436 |  |  |
|  | cln-miR156j | UGACAGAAGAAAGAGAGCAC | 20 | ath-miR156h | 427 |  |  |
|  | cln-miR156k | CUGACAGAAGAUAGAGAGCAC | 21 | smo-miR156b | 331 |  |  |
|  | cln-miR156l | UGACAGAGGAGAGUGAGCAC | 20 | vvi-miR156e | 286 |  |  |
|  | cln-miR156m | UGUCAGAAGAGAGUGAGCAC | 20 | ghr-miR156c | 156 |  |  |
|  | cln-miR156n | UGACAGAAGAGAGAGAGCACA | 21 | osa-miR156k | 10 |  |  |
|  | cln-miR156o | ACAGAAGAUAGAGAGCACAG | 20 | gma-miR156g | 1 |  |  |
|  | cln-miR157a | UUGACAGAAGAUAGAGAGCAC | 21 | ath-miR157a | 604506 | ++ |  |
|  | cln-miR157b | UGACAGAAGAUAGAGAGCAC | 20 | ath-miR157d | 36944 |  |  |
|  | cln-miR157c | UGACAGAAGAGAGAGAGCAU | 20 | vvi-miR156h | 8 |  |  |
| 158 | cln-miR158a | UCCCAAAUGUAGACAAAGCA | 20 | ath-miR158a | 14 | + |  |
| 159 | cln-miR159a | UUUGGAUUGAAGGGAGCUCUA | 21 | ath-miR159a | 2070 |  |  |
|  | cln-miR159b | UUUGGAUUGAAGGGAGCUCUU | 21 | ath-miR159b | 16 |  |  |
|  | cln-miR159c | CUUGGAUUGAAGGGAGCUCUA | 21 | osa-miR159f | 2 |  |  |
|  | cln-miR159d | UUUGGAUUGAAGGGAGCUCUG | 21 | osa-miR159a.1 | 1 |  |  |
|  | cln-miR159e | UUUGGACUGAAGGGAGCUCUA | 21 | aqc-miR159 | 1 |  |  |
|  | cln-miR319a | UUGGACUGAAGGGAGCUCCCU | 21 | ath-miR319a | 8 |  |  |
|  | cln-miR319b | UUGGACUGAAGGGAGCUCCC | 20 | mtr-miR319 | 1 |  |  |
| 160 | cln-miR160a | UGCCUGGCUCCCUGUAUGCCA | 21 | ath-miR160a | 49 |  |  |
| 161 | cln-miR161a | UCAAUGCAUUGAAAGUGACUA | 21 | ath-miR161.2 | 35 | + |  |
|  | cln-miR161b | UUGAAAGUGACUACAUCGGGG | 21 | aly-miR161.1 | 4460 |  |  |
| 162_1 | cln-miR162a | UCGAUAAACCUCUGCAUCCAG | 21 | ath-miR162a | 3922 |  |  |
| 162_2 | cln-miR162b | UCGAUAAACCUCUGCAUCCA | 20 | zma-miR162 | 28 |  |  |
|  | cln-miR162c | UCGAUAAACCUCUGCAUCCGG | 21 | bdi-miR162 | 1 |  |  |
|  | cln-miR162d | UUGAUAAACCUCUGCAUCCAG | 21 | ath-miR162a | 2 |  | 0.84 |
| 164 | cln-miR164a | UGGAGAAGCAGGGCACGUGCA | 21 | ath-miR164a | 13997 | ++ |  |
|  | cln-miR164b | UGGAGAAGCAGGGCACGUGCG | 21 | ath-miR164c | 1010 | ++ | 1.15 |
|  | cln-miR164c | UGGAGAAGCAGGGCACGUGCU | 21 | osa-miR164d | 304 |  |  |
|  | cln-miR164d | UGGAGAAGCAGGGUACGUGCA | 21 | osa-miR164c | 8 |  |  |
|  | cln-miR164e | UGGAGAAGCAGGGCACGUGAG | 21 | osa-miR164e | 1 |  |  |
| 165/166 | cln-miR165a | UCGGACCAGGCUUCAUCCCCC | 21 | ath-miR165a | 1563 | ++ |  |
|  | cln-miR165b | UCGGACCAGGCUUCAUCCCC | 20 | aly-miR165a | 36 |  |  |
|  | cln-miR166a | UCGGACCAGGCUUCAUUCCCC | 21 | ath-miR166a | 33285 | ++ |  |
|  | cln-miR166b | UCGGACCAGGCUUCAUUCCUC | 21 | osa-miR166g | 608 |  |  |
|  | cln-miR166c | UCGGACCAGGCUUCAUUCC | 19 | vvi-miR166a | 585 |  |  |
|  | cln-miR166d | UCGGACCAGGCUUCAUUCCC | 20 | zma-miR166h | 429 |  |  |
|  | cln-miR166e | UCGGACCAGGCUUCAUUCCCU | 21 | osa-miR166m | 37 |  |  |
|  | cln-miR166f | UCGGACCAGGCUUCAUUCCU | 20 | sbi-miR166k | 17 |  |  |
|  | cln-miR166g | UCGAACCAGGCUUCAUUCCCC | 21 | osa-miR166e | 12 |  |  |
|  | cln-miR166h | UCGGACCAGGCUUCAUUCCCCC | 22 | ctr-miR166 | 11 |  |  |
|  | cln-miR166i | CUCGGACCAGGCUUCAUUCCC | 21 | bdi-miR166e | 5 |  |  |
|  | cln-miR166j | UCGGACCAGGCUUCAUUCCUU | 21 | ptc-miR166n | 4 |  |  |
|  | cln-miR166k | UCGGGCCAGGCUUCAUCCCCC | 21 | mtr-miR166d | 2 |  |  |
|  | cln-miR166l | UCUCGGACCAGGCUUCAUUCC | 21 | bdi-miR166f | 2 |  |  |
|  | cln-miR166m | UCGGAUCAGGCUUCAUUCCUC | 21 | osa-miR166j | 1 |  |  |
|  | cln-miR166n | UCGGACCAGGCUUCAUUCCCUU | 22 | crt-miR166b | 1 |  |  |
|  | cln-miR166o | CCGGACCAGGCUUCAUCCCAG | 21 | pta-miR166c | 0 |  | 0.64 |
| 167_1 | cln-miR167a | UGAAGCUGCCAGCAUGAUCUA | 21 | ath-miR167a | 47800 |  |  |
|  | cln-miR167b | UGAAGCUGCCAGCAUGAUCUU | 21 | ptc-miR167f | 547 |  |  |
|  | cln-miR167c | UGAAGCUGCCAGCAUGAUCUGG | 22 | ath-miR167d | 85 |  |  |
|  | cln-miR167d | UGAAGCUGCCAGCAUGAUCUAA | 22 | bna-miR167a | 78 |  |  |
|  | cln-miR167e | UGAAGCUGCCAGCAUGAUCUC | 21 | vvi-miR167c | 60 |  |  |
|  | cln-miR167f | UGAAGCUGCCAGCAUGAUCUG | 21 | osa-miR167d | 48 |  |  |
|  | cln-miR167g | UGAAGCUGCCAGCAUGAUCUGA | 22 | ccl-miR167a | 40 |  |  |
|  | cln-miR167h | UGAAGCUGACAGCAUGAUCUA | 21 | tae-miR167b | 12 |  |  |
|  | cln-miR167i | UCAAGCUGCCAGCAUGAUCUA | 21 | aqc-miR167 | 4 |  |  |
| 167_2 | cln-miR167j | UAAGCUGCCAGCAUGAUCUUG | 21 | ath-miR167c | 48 |  |  |
| 168 | cln-miR168a | UCGCUUGGUGCAGGUCGGGAA | 21 | ath-miR168a | 9298 | + |  |
|  | cln-miR168b | UCGCUUGGUGCAGAUCGGGAC | 21 | osa-miR168a | 5 |  |  |
| 169_1 | cln-miR169a | UAGCCAAGGAUGACUUGCCUG | 21 | ath-miR169h | 1 | + |  |
| 169_2 | cln-miR169b | CAGCCAAGGAUGACUUGCCGA | 21 | ath-miR169a | 41 |  |  |
|  | cln-miR169c | UGAGCCAAGGAUGACUUGCCG | 21 | ath-miR169d | 26 |  |  |
|  | cln-miR169d | CAGCCAAGGAUGACUUGCCGG | 21 | ath-miR169b | 20 |  |  |
| 169_6 | cln-miR169e | UGAGCCAAAGAUGACUUGCCG | 21 | mtr-miR169i | 458 |  |  |
| 169_8 | cln-miR169f | AGCCAAGGAUGACUUGCCGG | 20 | gma-miR169e | 7 |  |  |
| 171_1 | cln-miR171a | UGAUUGAGCCGCGCCAAUAUC | 21 | ath-miR171a | 66 | ++ |  |
|  | cln-miR171b | UGAUUGAGCCGUGCCAAUAUC | 21 | osa-miR171b | 14 |  |  |
|  | cln-miR171c | UUGAGCCGUGCCAAUAUCAC | 20 | zma-miR171b | 11 |  |  |
|  | cln-miR171d | UUGAGCCGUGCCAAUAUCACG | 21 | ath-miR171b | 2 |  |  |
| 172 | cln-miR172a | AGAAUCUUGAUGAUGCUGCAU | 21 | ath-miR172a | 6593 | + |  |
|  | cln-miR172b | GAAUCUUGAUGAUGCUGCAU | 20 | aly-miR172e | 104 |  |  |
|  | cln-miR172c | GGAAUCUUGAUGAUGCUGCAU | 21 | ath-miR172e | 102 |  |  |
|  | cln-miR172d | AGAAUCUUGAUGAUGCUGCAG | 21 | ath-miR172c | 52 |  |  |
|  | cln-miR172e | AGAAUCUUGAUGAUGCUGCA | 20 | zma-miR172a | 25 |  |  |
|  | cln-miR172f | UGAGAAUCUUGAUGAUGCUGCAU | 23 | vvi-miR172d | 11 |  |  |
|  | cln-miR172g | AGAAUCCUGAUGAUGCUGCAU | 21 | tcc-miR172d | 5 |  |  |
| 390 | cln-miR390a | AAGCUCAGGAGGGAUAGCGCC | 21 | ath-miR390a | 596 | ++ |  |
| 391 | cln-miR391a | UUCGCAGGAGAGAUAGCGCCA | 21 | ath-miR391 | 2 |  |  |
| 393 | cln-miR393a | UCCAAAGGGAUCGCAUUGAUCC | 22 | ath-miR393a | 1 |  |  |
| 394 | cln-miR394a | UUGGCAUUCUGUCCACCUCC | 20 | ath-miR394a | 26 |  |  |
| 395 | cln-miR395a | CUGAAGUGUUUGGGGGGACUC | 21 | ath-miR395b | 123 |  |  |
|  | cln-miR395b | CUGAAGUGUUUGGGGGAACUC | 21 | ath-miR395a | 53 |  |  |
|  | cln-miR395c | UGAAGUGUUUGGGGGAACUC | 20 | tae-miR395b | 8 |  |  |
|  | cln-miR395d | UUGAAGUGUUUGGGGGAACUC | 21 | mtr-miR395g | 1 |  |  |
| 396 | cln-miR396a | UUCCACAGCUUUCUUGAACUG | 21 | ath-miR396a | 56 |  |  |
|  | cln-miR396b | UUCCACAGCUUUCUUGAACUU | 21 | ath-miR396b | 45 |  |  |
|  | cln-miR396c | UUCCACAGCUUUCUUGAACU | 20 | vvi-miR396b | 8 |  |  |
| 398 | cln-miR398a | UGUGUUCUCAGGUCACCCCUG | 21 | ath-miR398b | 1 |  |  |
|  | cln-miR398b | UGUGUUCUCAGGUCACCCCU | 20 | ahy-miR398 | 1 |  |  |
| 399 | cln-miR399a | UGCCAAAGGAGAGUUGCCCUG | 21 | ath-miR399b | 1 |  |  |
|  | cln-miR399b | UGCCAAAGGAGAUUUGCCCGG | 21 | ath-miR399f | 1 |  |  |
| 400 | cln-miR400a | UAUGAGAGUAUUAUAAGUCAC | 21 | ath-miR400 | 157 |  |  |
| 403 | cln-miR403a | UUAGAUUCACGCACAAACUCG | 21 | ath-miR403 | 184 |  |  |
| 408 | cln-miR408a | AUGCACUGCCUCUUCCCUGGC^*^ | 21 | ath-miR408 | 458 | ++ |  |
|  | cln-miR408b | UGCACUGCCUCUUCCCUGGCU | 21 | ppt-miR408b | 4 |  |  |
| 528 | cln-miR528a | UGGAAGGGGCAUGCAGAGGAG | 21 | osa-miR528 | 8 |  |  |
| 824 | cln-miR824a | UAGACCAUUUGUGAGAAGGGA | 21 | ath-miR824 | 1785 | + |  |
| 827_3 | cln-miR827a | UUAGAUGACCAUCAACAAACG | 21 | aly-miR827 | 901 |  |  |
|  | cln-miR827b | UUAGAUGACCAUCAACAAACU | 21 | ath-miR827 | 1 |  |  |
| 828 | cln-miR828a | UCUUGCUUAAAUGAGUAUUCCA | 22 | ath-miR828 | 1 |  |  |
| 845_1 | cln-miR845a | CGGCUCUGAUACCAAUUGAUG | 21 | ath-miR845a | 132 |  |  |
| 858 | cln-miR858a | UUUCGUUGUCUGUUCGACCUU | 21 | ath-miR858 | 2 |  |  |
| 894 | cln-miR894 | CGUUUCACGUCGGGUUCACC | 20 | ppt-miR894 | 89 | + |  |
| 1310 | cln-miR1310 | GGCAUCGGGGGCGUAACGCCCU | 22 | pta-miR1310 | 0 |  | 0.68 |
| 2111 | cln-miR2111a | UAAUCUGCAUCCUGAGGUUUA | 21 | ath-miR2111a | 1 |  |  |
| 5083 | cln-miR5083 | AGACUACAAUUAUCUGAUCA | 20 | osa-miR5083 | 1 |  |  |
| 5139 | cln-miR5139 | AAACCUGGCUCUGAUACCA | 19 | rgl-miR5139 | 18 |  |  |
|  | cln-tasiR2142^a^ | UUCUUGACCUUGUAAGACCCC | 21 |  | 566 | + |  |
|  | cln-siR1778^b^ | CUUGUAAGGCCUUUUCUUGAC | 21 |  | 4 |  |  |
|  | cln-siR1769^b^ | UUCUCCUACCUUGUCUAUCCC | 21 |  | 9 |  |  |
| N1 | cln-miRn1 | UGGCAUCUGUCGAGGUCAUCUA | 22 |  | 17 | ++ |  |
| ‘+’ indicates that the mature miRNA was detected by RT-PCR, ‘++’ means the miRNA was further validated by subcloning.  ^a^ identical to *A. thaliana* TAS3a D7(+).  ^b^ identical to the siRNA counterpart of *A. thaliana*. | | | | | | | |
