# Supplementary material for: Identification and characterization of small non-coding RNAs from Chinese fir by high throughput sequencing
Source: BMC Plant Biol. 2012 Aug 15;12:146. doi: 10.1186/1471-2229-12-146 (PMC3462689; doi:10.1186/1471-2229-12-146)
Supplement: Additional file 3 — Precursor and primary sequences of conserved and novel miRNAs in Chinese fir. [file 1471-2229-12-146-S3.doc]

**Additional file 3 Precursor and primary sequences of conserved and novel miRNAs in Chinese fir.**

| **Name** | **Sequence (5'-3')** | **Length (nt)** |
| --- | --- | --- |
| **pre-miR162d** | UUAACUGGAGGCAGGGUUUUAUCCAUCCCUUCCUGUUACUGAUUUAAGGCUCUGCUCACAAUUAACUGCAGUUAACAGGCACGGGUUGAUAAACCUCUGCAUCCAGUUUGG | 111 |
| **pre-miR164b** | CCUGGUGGAGAAGCAGGGCACGUGCGAUACACAGAAACAAUUGUAUAAACCAUUAUCGCAUGUGCUUUCCUUCUCCACCAGGC | 83 |
| **pre-miR166o** | UCCAAUCGAAUCCGGACCAGGCUUCAUCCCAGGCAUCUGGACCCAAUCGACAGCAGUUCCUGUAGCCUUUAAAAGGAACUCUGCCAAGGUCUCCUCUGCAAUAGACAGGAGUCCAGCGGGGCUAGCAUCCCUGAGGGGAUGCUGAGGUGUUGGGUUGUGUUGGU | 164 |
| **pre-miR1310** | AGGCAUCGGGGGCGUAACGCCCUCGACCUAUUCUCAAACUUUAAAUAGGUAAGGCGGCGCGGCUGCUCCGUUGAGCCGC | 79 |
| **pre-miRn1** | GGUAGAUUCUUGGCAUCUGUCGAGGUCAUCUACAAAGUUUUCGUUUGUUGAUAUUAGAAAAAUAUAAAUUUUAGCAAGUGGAUCUAAAACAUUGUGGAUGAUCUCGAGGAUGCCCAAGGGUUUAUUA | 127 |
| **pri-miRn1 (Unigene 1397)** | CAAAUGGUUUAUUUUUCCGUGAUCUCUCCCCUCUUAUCGCUUUGGCAUUUGAUAUCUAUAAAUUAGCGUUCAGGGAUAAGAGUGGCGUGGUUUUUCGUUGGCGUUAUACAUACGGUGAGAGGAUCUAGGGUUCGAGUGCGAUACGGGGAAGAACGCAGAGUGGUAGAUUCUUGGCAUCUGUCGAGGUCAUCUACAAAGUUUUCGUUUGUUGAUAUUAGAAAAAUAUAAAUUUUAGCAAGUGGAUCUAAAACAUUGUGGAUGAUCUCGAGGAUGCCCAAGGGUUUAUUACUUUGCAGGCAUAUUCAGAGAUGUUUAUUUGUGUUUAGCAGGUUUGAUUGCUAGAGUUGGCACGUAUAAGAAAAACCCUUGUUAUCUGUUUGUUGCAGUUUUAAGUUUAUUCUUUUUAUUUGUUUCAGACGAUUCAGGAUGAUGUUCUCCUGAAAUUUAUAAUAUAAGUGCUUUUCUUUUGGAUGCAUCUUUGUUUAUAAAAUUUGGAGCUGCCAUUGGUUCCUCAGAUUCUGAUAUUAGAUCUGUGGGUCUGCCUCUGUUUCCUGACACCAAAUUUGUUAGUUUUAGUAGUUGCAAAGUUUCAGCUGAAUAUAGACGGAGUGCCUUUUCAGUUGGGGCCUCGUUAUCGUUUCUGUUAUAGUAAUCGAGUAUGGUAAAUGAGAUUCUGUCGUUAUUGGUUUGCCUGAGACUCUAAGAUUUGGAGUUCUAUUUUUGCUUUUGGGUUUUCAGGGUUUCAUCUGUUUUUUUGACGGUGAAAUCAUUUCCACCAGGCUAUAUGGUAGAUCUUUUUAUACCUUUAAUGUAAAGGGUGUUUUUUUGGGGG | 832 |
