# Supplementary figures and images for: Identification and characterization of small non-coding RNAs from Chinese fir by high throughput sequencing
Source: BMC Plant Biol. 2012 Aug 15;12:146. doi: 10.1186/1471-2229-12-146 (PMC3462689; doi:10.1186/1471-2229-12-146)

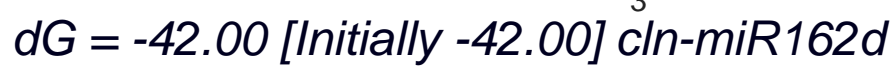

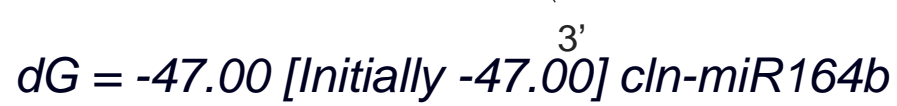

$dG = -47.00$  [Initially -47.00] *cln-miR164b*

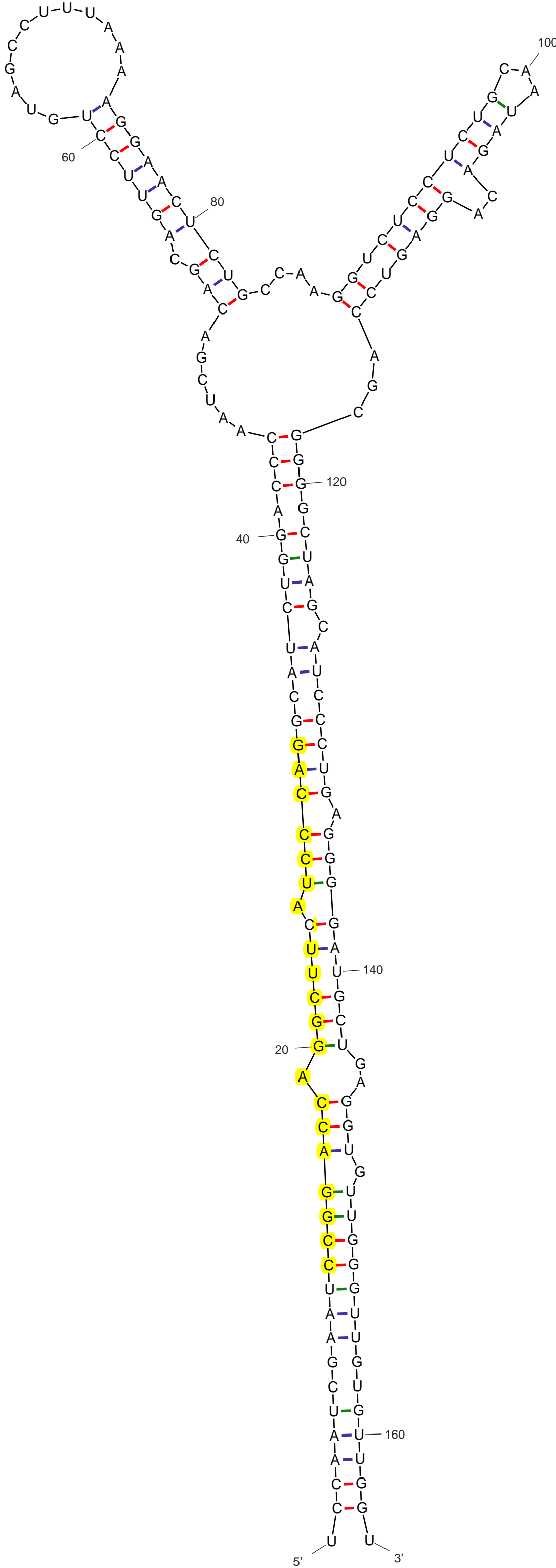

*dG = -57.65 [Initially -61.60] cln-miR166a*

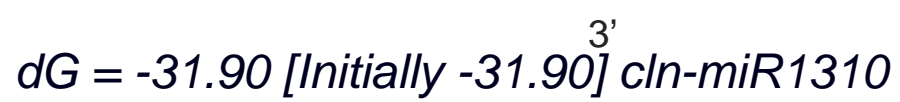
$$dG = -31.90 \text{ [Initially } -31.90] \text{ } ^3\text{cln-miR1310}$$

Supplement: Additional file 4 — The hairpin structures of conserved and novel miRNAs predicted by MFOLD. [file 1471-2229-12-146-S4.pdf]

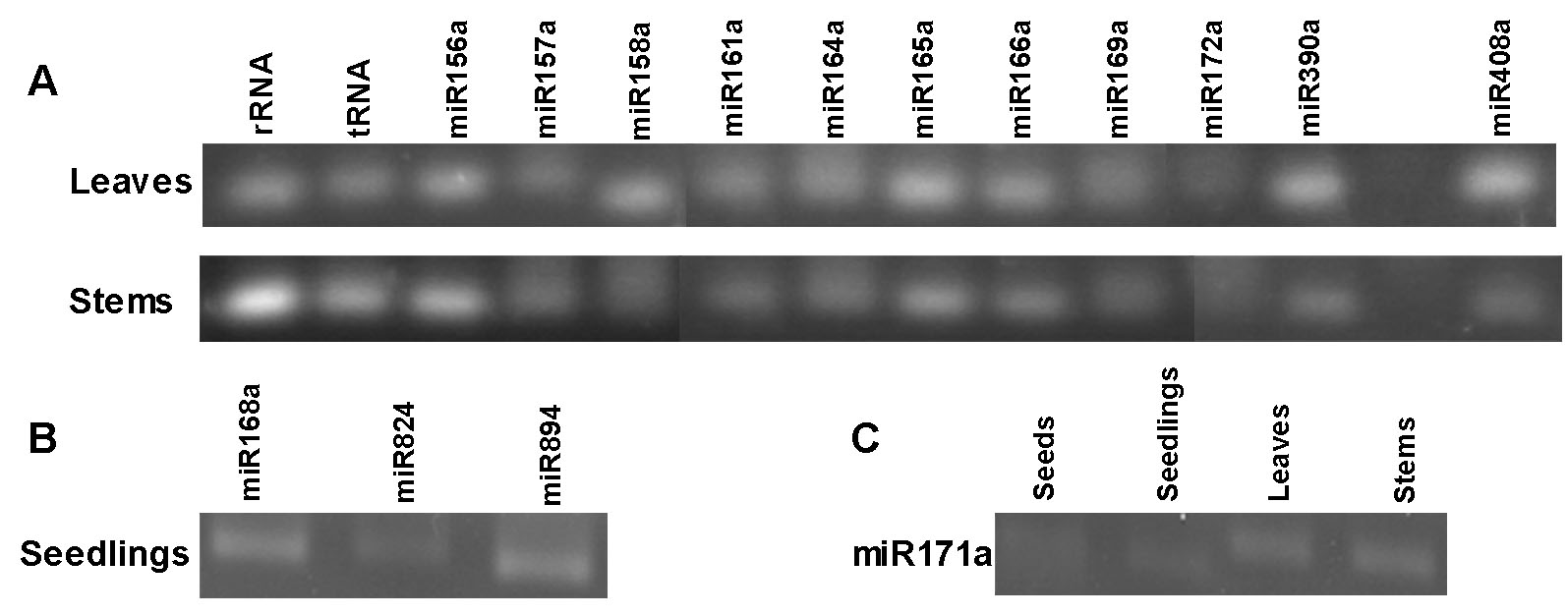

Supplement: Additional file 5 — RT-PCR of conserved mature miRNAs. [file 1471-2229-12-146-S5.jpeg]

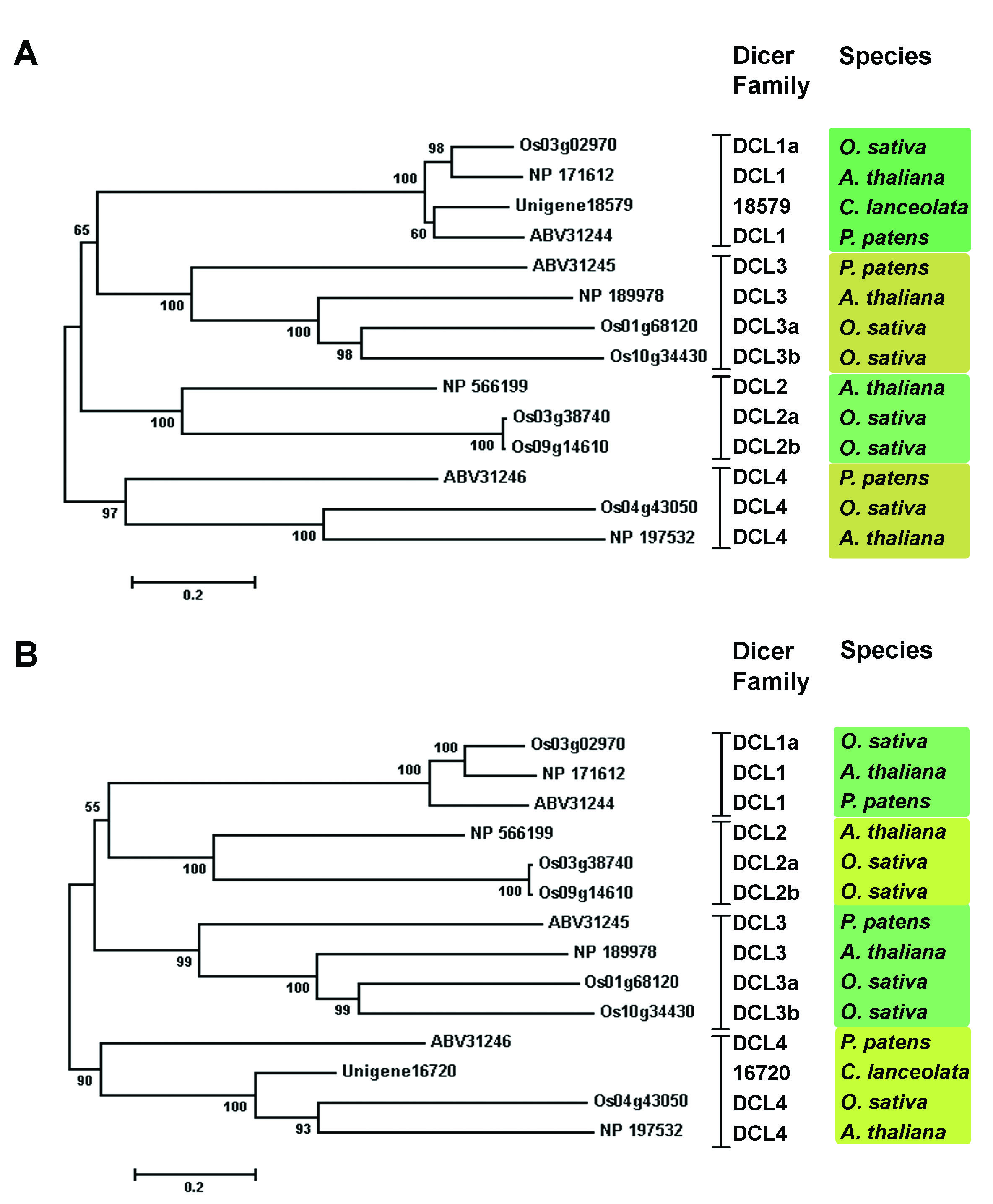

Supplement: Additional file 9 — Phylogenetic trees of unigene18579 and unigene16720 with three model plant DCLs. [file 1471-2229-12-146-S9.jpeg]
