# Supplementary material for: Identification and characterization of small non-coding RNAs from Chinese fir by high throughput sequencing
Source: BMC Plant Biol. 2012 Aug 15;12:146. doi: 10.1186/1471-2229-12-146 (PMC3462689; doi:10.1186/1471-2229-12-146)
Supplement: Additional file 6 — Unigenes involved in the biogenesis and action of miRNAs in Chinese fir. [file 1471-2229-12-146-S6.doc]

**Additional file 6 Unigenes involved in the biogenesis and action of miRNAs in Chinese fir.**

| **Gene name** | **Unigene annotation** | **Unigene** | **Conserved in other plants** | **E-score** |
| --- | --- | --- | --- | --- |
| *Dicer-like1* (*DCL1*) | DCL1 | Unigene16794 | *Arabidopsis thaliana* | 4.00E-42 |
|  |  | Unigene18579 | *Arabidopsis thaliana* | 0 |
|  |  | Unigene22262 | *Arabidopsis thaliana* | 2.00E-08 |
|  |  | Unigene34518 | *Arabidopsis thaliana* | 4.00E-08 |
|  |  | Unigene37365 | *Arabidopsis thaliana* | 1.00E-20 |
|  |  | Unigene40285 | *Arabidopsis thaliana* | 8.00E-19 |
|  |  | Unigene41759 | *Arabidopsis thaliana* | 2.00E-06 |
|  |  | Unigene44442 | *Arabidopsis thaliana* | 2.00E-14 |
|  |  | Unigene45244 | *Arabidopsis thaliana* | 4.00E-09 |
|  |  | Unigene48030 | *Arabidopsis thaliana* | 4.00E-59 |
|  |  | Unigene48281 | *Arabidopsis thaliana* | 2.00E-32 |
|  |  | Unigene51372 | *Arabidopsis thaliana* | 1.00E-60 |
|  |  | Unigene52674 | *Arabidopsis thaliana* | 1.00E-18 |
|  |  | Unigene53195 | *Arabidopsis thaliana* | 8.00E-91 |
|  |  | Unigene5165 | *Arabidopsis thaliana* | 9.00E-64 |
|  |  | Unigene5851 | *Arabidopsis thaliana* | 1.00E-29 |
|  | Endoribonuclease Dicer homolog 1 | Unigene37365 | *Oryza sativa* | 1.00E-20 |
|  |  | Unigene16794 | *Oryza sativa* | 1.00E-39 |
|  |  | Unigene18579 | *Oryza sativa* | 0 |
|  |  | Unigene22262 | *Oryza sativa* | 5.00E-08 |
|  |  | Unigene29012 | *Oryza sativa* | 3.00E-09 |
|  |  | Unigene34518 | *Oryza sativa* | 1.00E-07 |
|  |  | Unigene37365 | *Oryza sativa* | 1.00E-21 |
|  |  | Unigene40285 | *Oryza sativa* | 7.00E-20 |
|  |  | Unigene40957 | *Oryza sativa* | 4.00E-07 |
|  |  | Unigene44442 | *Oryza sativa* | 2.00E-14 |
|  |  | Unigene45244 | *Oryza sativa* | 4.00E-08 |
|  |  | Unigene47836 | *Oryza sativa* | 1.00E-14 |
|  |  | Unigene48030 | *Oryza sativa* | 4.00E-36 |
|  |  | Unigene48281 | *Oryza sativa* | 7.00E-34 |
|  |  | Unigene51372 | *Oryza sativa* | 6.00E-60 |
|  |  | Unigene52674 | *Oryza sativa* | 5.00E-18 |
|  |  | Unigene53195 | *Oryza sativa* | 1.00E-89 |
|  |  | Unigene4521 | *Oryza sativa* | 1.00E-24 |
|  |  | Unigene5165 | *Oryza sativa* | 3.00E-67 |
|  |  | Unigene5851 | *Oryza sativa* | 5.00E-30 |
|  |  | Unigene11609 | *Oryza sativa* | 5.00E-06 |
| *Argonaute1* (*AGO1*) | AGO1 | Unigene16266 | *Arabidopsis thaliana* | 1.00E-98 |
|  |  | Unigene18547 | *Arabidopsis thaliana* | 0 |
|  |  | Unigene35230 | *Arabidopsis thaliana* | 7.00E-07 |
|  |  | Unigene51924 | *Arabidopsis thaliana* | 8.00E-83 |
|  |  | Unigene58812 | *Arabidopsis thaliana* | 0 |
|  |  | Unigene59402 | *Arabidopsis thaliana* | 0 |
|  |  | Unigene6526 | *Arabidopsis thaliana* | 0 |
|  |  | Unigene8967 | *Arabidopsis thaliana* | 1.00E-160 |
|  |  | Unigene16266 | *Arabidopsis thaliana* | 1.00E-98 |
|  |  | Unigene18547 | *Arabidopsis thaliana* | 0 |
|  |  | Unigene35230 | *Arabidopsis thaliana* | 7.00E-07 |
|  |  | Unigene51924 | *Arabidopsis thaliana* | 8.00E-83 |
|  |  | Unigene58812 | *Arabidopsis thaliana* | 0 |
|  |  | Unigene59402 | *Arabidopsis thaliana* | 0 |
|  |  | Unigene59409 | *Arabidopsis thaliana* | 1.00E-68 |
|  |  | Unigene6526 | *Arabidopsis thaliana* | 0 |
|  |  | Unigene8967 | *Arabidopsis thaliana* | 1.00E-162 |
|  | AGO1-1 | Unigene15418 | *Nicotiana benthamiana* | 2.00E-07 |
|  |  | Unigene16266 | *Nicotiana benthamiana* | 1.00E-102 |
|  |  | Unigene18547 | *Nicotiana benthamiana* | 0 |
|  |  | Unigene35230 | *Nicotiana benthamiana* | 9.00E-07 |
|  |  | Unigene40503 | *Nicotiana benthamiana* | 8.00E-35 |
|  |  | Unigene50056 | *Nicotiana benthamiana* | 6.00E-28 |
|  |  | Unigene51924 | *Nicotiana benthamiana* | 3.00E-84 |
|  |  | Unigene58812 | *Nicotiana benthamiana* | 0 |
|  |  | Unigene59402 | *Nicotiana benthamiana* | 0 |
|  |  | Unigene59409 | *Nicotiana benthamiana* | 7.00E-73 |
|  |  | Unigene6526 | *Nicotiana benthamiana* | 0 |
|  |  | Unigene8967 | *Nicotiana benthamiana* | 1.00E-175 |
|  | AGO1-2 | Unigene15418 | *Nicotiana benthamiana* | 1.00E-08 |
|  |  | Unigene16266 | *Nicotiana benthamiana* | 2.00E-99 |
|  |  | Unigene18547 | *Nicotiana benthamiana* | 0 |
|  |  | Unigene35230 | *Nicotiana benthamiana* | 9.00E-07 |
|  |  | Unigene50056 | *Nicotiana benthamiana* | 1.00E-27 |
|  |  | Unigene51924 | *Nicotiana benthamiana* | 3.00E-87 |
|  |  | Unigene58812 | *Nicotiana benthamiana* | 0 |
|  |  | Unigene59402 | *Nicotiana benthamiana* | 0 |
|  |  | Unigene59409 | *Nicotiana benthamiana* | 2.00E-70 |
|  |  | Unigene6526 | *Nicotiana benthamiana* | 0 |
|  |  | Unigene8967 | *Nicotiana benthamiana* | 1.00E-171 |
|  | AGO1A | Unigene16266 | *Oryza sativa* | 6.00E-99 |
|  |  | Unigene18547 | *Oryza sativa* | 0 |
|  |  | Unigene31751 | *Oryza sativa* | 7.00E-12 |
|  |  | Unigene40503 | *Oryza sativa* | 2.00E-34 |
|  |  | Unigene50056 | *Oryza sativa* | 2.00E-27 |
|  |  | Unigene51924 | *Oryza sativa* | 2.00E-82 |
|  |  | Unigene58812 | *Oryza sativa* | 0 |
|  |  | Unigene59402 | *Oryza sativa* | 0 |
|  |  | Unigene6526 | *Oryza sativa* | 0 |
|  |  | Unigene8967 | *Oryza sativa* | 1.00E-168 |
|  |  | Unigene18547 | *Oryza sativa* | 0 |
|  |  | Unigene35230 | *Oryza sativa* | 7.00E-07 |
|  |  | Unigene40503 | *Oryza sativa* | 1.00E-34 |
|  |  | Unigene51924 | *Oryza sativa* | 2.00E-80 |
|  |  | Unigene59402 | *Oryza sativa* | 0 |
|  |  | Unigene59409 | *Oryza sativa* | 5.00E-70 |
|  |  | Unigene6526 | *Oryza sativa* | 0 |
|  |  | Unigene8967 | *Oryza sativa* | 1.00E-158 |
|  |  | Unigene18547 | *Oryza sativa* | 1.00E-179 |
|  |  | Unigene35230 | *Oryza sativa* | 2.00E-07 |
|  |  | Unigene51924 | *Oryza sativa* | 4.00E-79 |
|  |  | Unigene59402 | *Oryza sativa* | 0 |
|  |  | Unigene6526 | *Oryza sativa* | 0 |
|  |  | Unigene8967 | *Oryza sativa* | 1.00E-158 |
|  |  | Unigene31751 | *Oryza sativa* | 7.00E-12 |
|  |  | Unigene49081 | *Oryza sativa* | 6.00E-06 |
|  |  | Unigene54975 | *Oryza sativa* | 4.00E-49 |
| *HEN1* | HEN1 | Unigene39786 | *Arabidopsis thaliana* | 9.00E-07 |
|  |  | Unigene8390 | *Arabidopsis thaliana* | 2.00E-29 |
| *DNA-dependent RNA polymeraseII*  (*Pol II*) | DNA-dependent RNA polymerase II | Unigene41476 | *Arabidopsis thaliana* | 4.00E-12 |
|  |  | Unigene42360 | *Arabidopsis thaliana* | 1.00E-09 |
|  |  | Unigene12438 | *Arabidopsis thaliana* | 0 |
|  | DNA-dependent RNA polymerase II largest subunit | Unigene42235 | *Cyanophora paradoxa* | 3.00E-22 |
|  |  | Unigene42235 | Cyanophora paradoxa | 3.00E-22 |
|  |  | Unigene15095 | *Glaucocystis nostochinearum* | 1.00E-47 |
|  | DNA-directed RNA polymerase II second largest subunit | Unigene32251 | *Guillardia theta* | 2.00E-08 |
|  |  | Unigene8812 | *Guillardia theta* | 9.00E-41 |
|  | DNA-directed RNA polymerase II third largest subunit | Unigene42400 | *Medicago sativa* | 4.00E-19 |
|  | RNA polymerase II fifth largest subunit like protein | Unigene42168 | *Arabidopsis thaliana* | 1.00E-10 |
|  |  | Unigene7829 | *Arabidopsis thaliana* | 3.00E-33 |
|  |  | Unigene42168 | *Arabidopsis thaliana* | 2.00E-12 |
|  |  | Unigene7829 | *Arabidopsis thaliana* | 2.00E-29 |
| *Hyponastic leaves1* (*HYL1*) | HYL1 | Unigene1587 | *Arabidopsis thaliana* | 3.00E-10 |
|  |  | Unigene49025 | *Arabidopsis thaliana* | 3.00E-09 |
|  |  | Unigene52857 | *Arabidopsis thaliana* | 5.00E-31 |
| *Serrate* (*SE*) | SE | Unigene51981 | *Arabidopsis thaliana* | 2.00E-27 |
|  |  | Unigene56418 | *Arabidopsis thaliana* | 4.00E-79 |
|  |  | Unigene57153 | *Arabidopsis thaliana* | 1.00E-101 |
| *Dawdle* (*DDL*) | DDL | Unigene15802 | *Arabidopsis thaliana* | 9.00E-90 |
|  |  | Unigene19081 | *Arabidopsis thaliana* | 1.00E-07 |
|  |  | Unigene12813 | *Arabidopsis thaliana* | 7.00E-09 |
| *HASTY* | HASTY | Unigene16778 | *Arabidopsis thaliana* | 1.00E-28 |
|  |  | Unigene17280 | *Arabidopsis thaliana* | 2.00E-64 |
|  |  | Unigene18043 | *Arabidopsis thaliana* | 1.00E-84 |
|  |  | Unigene22530 | *Arabidopsis thaliana* | 2.00E-13 |
|  |  | Unigene39063 | *Arabidopsis thaliana* | 7.00E-18 |
|  |  | Unigene2107 | *Arabidopsis thaliana* | 3.00E-20 |
|  |  | Unigene16778 | *Arabidopsis thaliana* | 1.00E-28 |
|  |  | Unigene17280 | *Arabidopsis thaliana* | 2.00E-64 |
|  |  | Unigene18043 | *Arabidopsis thaliana* | 4.00E-85 |
|  |  | Unigene22530 | *Arabidopsis thaliana* | 2.00E-13 |
|  |  | Unigene39063 | *Arabidopsis thaliana* | 7.00E-18 |
|  |  | Unigene2107 | *Arabidopsis thaliana* | 3.00E-20 |
