# Supplementary material for: Identification and characterization of small non-coding RNAs from Chinese fir by high throughput sequencing
Source: BMC Plant Biol. 2012 Aug 15;12:146. doi: 10.1186/1471-2229-12-146 (PMC3462689; doi:10.1186/1471-2229-12-146)
Supplement: Additional file 7 — Conserved and novel miRNA targets and their putative functions. [file 1471-2229-12-146-S7.doc]

**Additional file 7 Conserved and novel miRNA targets and their putative functions.**

| **miRNA family** | **Target unigene** | **Score** | **Annotation** | **E-value** |
| --- | --- | --- | --- | --- |
| cln-MIR156/157 | Unigene10188 | 3 | LNG1 (Longifolia1) [*Arabidopsis thaliana*] | 1.0E-24 |
|  | Unigene12464 | 3 | Sugar isomerase (SIS) domain-containing protein [*Arabidopsis thaliana*] | 2.0E-09 |
|  | Unigene12489 | 3 | Chorion family 2 protein [*Oryza sativa*] | 2.0E-10 |
|  | Unigene12536 | 3 | Short-chain dehydrogenase/reductase family protein [*Arabidopsis lyrata*] | 3.0E-53 |
|  | Unigene13706 | 2.5 | RNA recognition motif family protein [*Oryza sativa*] | 4.0E-52 |
|  | Unigene13959 | 3 | Allantoate amidohydrolase [*Glycine max*] | 1.0E-159 |
|  | Unigene14603 | 3 | Amino acid transporter [*Populus trichocarpa*] | 1.0E-151 |
|  | Unigene16419 | 3 | Cationic amino acid transmembrane transporter [*Arabidopsis thaliana*] | 1.0E-147 |
|  | Unigene17397 | 3 | Agenet domain-containing protein [*Arabidopsis lyrata* subsp. lyrata] | 5.0E-24 |
|  | Unigene18038 | 3 | Homogentisate 1,2-dioxygenase [*Solanum lycopersicum*] | 0.0E+00 |
|  | Unigene18862 | 3 | Delta-1-pyrroline-5-carboxylate dehydrogenase 12A1 [*Arabidopsis thaliana*] | 0.0E+00 |
|  | Unigene2030 | 2 | SBP-domain protein 13 [*Physcomitrella patens*] | 2.0E-61 |
|  | Unigene2108 | 3 | Unknown |  |
|  | Unigene21828 | 2.5 | Unknown |  |
|  | Unigene27673 | 2 | Monooxygenase family protein [*Arabidopsis thaliana*] | 6.0E-07 |
|  | Unigene2872 | 2 | Squamosa promoter-binding-like protein 7 [*Oryza sativa*] | 9.0E-39 |
|  | Unigene29354 | 3 | Axi 1 protein [*Arabidopsis thaliana*] | 4.0E-29 |
|  | Unigene36905 | 3 | Unknown |  |
|  | Unigene37124 | 3 | Unknown |  |
|  | Unigene43286 | 3 | Unknown |  |
|  | Unigen45299 | 3 | Unknown |  |
|  | Unigene46795 | 3 | Leucine Rich Repeat family protein [*Oryza sativa*] | 1.0E-07 |
|  | Unigene47711 | 2 | Unknown |  |
|  | Uingen4854 | 3 | Unknown |  |
|  | Unigene48754 | 3 | Unknown |  |
|  | Unigene49281 | 3 | Plus agglutinin [*Chlamydomonas reinhardtii*] | 1.0E-06 |
|  | Unigene50522 | 3 | Leucine-rich repeat protein kinase [*Populus trichocarpa*] | 2.0E-14 |
|  | Unigene50931 | 3 | Unknown |  |
|  | Unigene52061 | 3 | RbohF [*Nicotiana tabacum*] | 9.0E-50 |
|  | Unigene54166 | 2.5 | tRNA synthetase class II (G, H, P and S) family protein [*Arabidopsis thaliana*] | 4.0E-63 |
|  | Unigene55364 | 3 | Unknown |  |
|  | Unigene55494 | 2.5 | Transcription factor LHY [*Populus nigra*] | 2.0E-10 |
|  | Unigene56735 | 3 | Agenet domain-containing protein / bromo-adjacent homology (BAH) domain-containing protein [*Arabidopsis thaliana*] | 1.0E-39 |
|  | Unigene57989 | 2.5 | PMR5 (POWDERY MILDEW RESISTANT 5) [*Arabidopsis thaliana*] | 1.0E-69 |
|  | Unigene5822 | 3 | Ankyrin repeat family protein [*Arabidopsis thaliana*] | 1.0E-32 |
|  | Unigene58554 | 3 | Unknown |  |
|  | Unigene58579 | 3 | Eukaryotic translation initiation factor 3 subunit A [*Arabidopsis thaliana*] | 1.0E-159 |
|  | Unigene59593 | 2.5 | CwfJ-like family protein [*Arabidopsis thaliana*] | 0.0E+00 |
|  | Unigene8339 | 3 | Impaired sucrose induction 1-like protein [*Medicago truncatula*] | 2.0E-69 |
|  | Unigene9100 | 3 | COP1 [*Oryza sativa* Japonica Group] | 0.0E+00 |
| cln-MIR159/319 | Unigene10970 | 3 | Zinc finger family protein [*Arabidopsis lyrata*] | 2.0E-38 |
|  | Unigene1099 | 3 | Oxidoreductase/ transition metal ion binding protein [*Arabidopsis lyrata*] | 1.0E-65 |
|  | Unigene18565 | 3 | Unknown [*Arabidopsis lyrata*] | 1.0E-118 |
|  | Unigene34804 | 3 | Unknown |  |
|  | Unigene3675 | 3 | Mitochondrial transcription termination factor family protein [*Arabidopsis lyrata* | 8.0E-23 |
|  | Unigene44010 | 3 | LAF3 isoform 1 [*Arabidopsis thaliana*] | 5.0E-18 |
|  | Unigene47678 | 3 | NBS/LRR [*Pinus taeda*] | 6.0E-19 |
|  | Unigene47729 | 3 | Disease resistance protein Cf-2.1-like [*Oryza sativa*] | 1.0E-10 |
|  | Unigene56017 | 3 | Unknown |  |
|  | Unigene58796 | 2.5 | Serine/threonine-protein phosphatase BSL3 [*Arabidopsis thaliana*] | 3.0E-150 |
|  | Unigene59220 | 3 | F-box/ankyrin repeat protein SKIP35 [*Arabidopsis thaliana*] | 2.0E-38 |
|  | Unigene7195 | 3 | ATP binding / microtubule motor [*Arabidopsis thaliana*] | 2.0E-28 |
| cln-MIR161 | Unigene10682 |  | Unknown |  |
|  | Unigene19617 | 3 | tRNA (guanine-N7-)-methyltransferase [*Arabidopsis thaliana*] | 4.0E-08 |
|  | Unigene51376 | 3 | Ribosomal protein S14p/S29e containing protein [*Oryza sativa*] | 5.0E-11 |
|  | Unigene55340 | 2.5 | Rab5/RabF-family small GTPase [*Physcomitrella patens*] | 6.0E-67 |
|  | Unigene7680 | 3 | Unknown |  |
| cln-MIR162 | Unigene13835 | 3 | Hydrolase [*Arabidopsis thaliana*] | 2.0E-46 |
|  | Unigene15004 | 2.5 | Unknown [*Arabidopsis thaliana*] | 2.0E-21 |
|  | Unigene44038 | 3 | Unknown |  |
| cln-MIR164 | Unigene11154 | 3 | RNA binding / catalytic protein [*Arabidopsis thaliana*] | 5.0E-91 |
|  | Unigene12718 | 1.5 | Unknown |  |
|  | Unigene38164 | 3 | Pentatricopeptide repeat-containing protein [*Arabidopsis lyrata*] | 6.0E-09 |
|  | Unigene4389 | 3 | GALT1 (GALACTOSYLTRANSFERASE1) [*Arabidopsis thaliana*] | 1.0E-27 |
|  | Unigene45355 | 3 | Unknown |  |
|  | Unigene49668 | 3 | VAP27 [*Zea mays*] | 3.0E-51 |
|  | Unigene50230 | 3 | Unknown |  |
|  | Unigene53405 | 3 | UXS4 (UDP-XYLOSE SYNTHASE 4) [*Arabidopsis thaliana*] | 1.0E-108 |
|  | Unigene58710 | 2.5 | ATP binding protein [*Zea mays*] | 3.0E-55 |
|  | Unigene6011 | 3 | Cytochrome p450 [*Pinus radiata*] | 1.0E-10 |
|  | Unigene7992 | 3 | Pentatricopeptide (PPR) repeat-containing protein-like [*Oryza sativa*] | 2.0E-21 |
|  | Unigene9075 | 3 | Calmodulin binding protein [*Zea mays*] | 4.0E-49 |
| cln-MIR166 | Unigene57974 | 3 | Glycosyl hydrolase family 9 [*Populus trichocarpa*] | 1.0E-142 |
|  | Unigene1626 | 3 | Kinase family protein [*Arabidopsis lyrata*] | 2.0E-54 |
|  | Unigene9280 | 3 | Auxin response factor 2 [*Cucumis sativus*] | 6.0E-49 |
|  | Unigene6787 | 3 | Glycosyltransferase, CAZy family GT90 [*Selaginella moellendorffii*] | 4.0E-20 |
|  | Unigene2120 | 3 | DTA2 (DOWNSTREAM TARGET OF AGL15 2) [*Arabidopsis thaliana*] | 3.0E-71 |
| cln-MIR167 | Unigene11885 | 3 | SAP domain containing protein [*Oryza sativa*] | 7.0E-95 |
|  | Unigene19102 | 3 | Leucine-rich repeat protein-related [*Arabidopsis thaliana*] | 0.0E+00 |
|  | Unigene28771 | 3 | Unknown |  |
|  | Unigene52928 | 3 | Ferredoxin-dependent glutamate synthase [*Glycine max*] | 2.0E-99 |
|  | Unigene56859 | 3 | Unknown [*Oryza sativa*] | 7.0E-25 |
|  | Unigene58257 | 3 | Electron transfer flavoprotein beta-subunit-like [*Arabidopsis thaliana*] | 1.0E-105 |
|  | Unigene7717 | 3 | TIR/NBS [*Pinus taeda*] | 8.0E-11 |
| cln-MIR168 | Unigene11614 | 3 | Unknown |  |
|  | Unigene6526 | 3 | Argonaute protein group [*Populus trichocarpa*] | 0.0E+00 |
|  | Unigene55099 | 3 | Proton pump interactor [*Arabidopsis thaliana*] | 6.0E-23 |
| cln-MIR169 | Unigene18994 | 3 | Protein kinase family protein [*Arabidopsis thaliana*] | 0.0E+00 |
|  | Unigene13175 | 3 | F-box family protein [*Arabidopsis lyrata*] | 3.0E-66 |
| cln-MIR171 | Unigene13248 | 2 | GHMP kinase family protein [*Arabidopsis lyrata*] | 1.0E-128 |
| cln-MIR172 | Unigene10194 | 2.5 | F-box family protein [*Populus trichocarpa*] | 1.0E-115 |
|  | Unigene10319 | 3 | HB09p [*Malus floribunda*] | 3.0E-13 |
|  | Unigene10617 | 3 | Unknown |  |
|  | Unigene14666 | 3 | Unknown |  |
|  | Unigene11698 | 3 | SET domain protein [*Populus trichocarpa*] | 0.0E+00 |
|  | Unigene14546 | 2.5 | Pentatricopeptide (PPR) repeat-containing protein [*Arabidopsis thaliana*] | 7.0E-15 |
|  | Unigene17250 | 2.5 | WD-40 repeat family protein [*Arabidopsis lyrata*] | 3.0E-51 |
|  | Unigene17425 | 2.5 | APETALA2 L2 [*Larix x marschlinsii*] | 1.0E-22 |
|  | Unigene17593 | 2.5 | SCARECROW [*Pinus sylvestris*] | 0.0E+00 |
|  | Unigene18581 | 3 | HEAT repeat family protein[*Oryza sativa*] | 1.0E-135 |
|  | Unigene18601 | 2.5 | Unknown [*Oryza sativa*] | 7.0E-75 |
|  | Unigene2805 | 3 | SCL28; RNA binding / nucleic acid binding / nucleotide binding [*Arabidopsis thaliana*] | 3.0E-47 |
|  | Unigene34088 | 3 | Pentatricopeptide repeat-containing protein [*Arabidopsis lyrata*] | 5.0E-16 |
|  | Unigene35426 | 2.5 | Unknown |  |
|  | Unigene36280 | 3 | Phytoalexin-deficient 4-2 protein [*Solanum tuberosum*] | 6.0E-08 |
|  | Unigene44006 |  | Unknown |  |
|  | Unigene46009 |  | Unknown |  |
|  | Unigene50436 |  | NADH ubiquinone oxidoreductase B14 subunit [*Zea mays*] | 3.0E-21 |
|  | Unigene54680 |  | Unknown |  |
|  | Unigene56981 | 1.5 | APETALA2-like protein 2 [*Pinus thunbergii*] | 1.0E-45 |
|  | Unigene8332 | 2.5 | Os06g0186600 [*Oryza sativa*] | 5.0E-85 |
|  | Unigene9067 | 2.5 | Polynucleotide adenylyltransferase [*Arabidopsis lyrata*] | 1.0E-71 |
|  | Unigene7755 | 3 | Zinc ion binding protein [*Arabidopsis thaliana*] | 3.0E-17 |
| cln-MIR390 | Unigene59587 | 2 | BRL1 (BRI 1 LIKE); kinase [*Arabidopsis thaliana*] | 0.0E+00 |
|  | Unigene18887 | 1 | Unknown |  |
|  | Unigene49603 | 2 | Unknown |  |
|  | Unigene51265 | 1.5 | Unknown |  |
|  | Unigene43272 | 3 | Helicase associated domain family protein [*Oryza sativa*] | 4.0E-34 |
| cln-MIR391 | Unigene6271 | 3 | Pectase lyase [*Prunus persica*] | 1.0E-119 |
| cln-MIR394 | Unigene15289 | 2.5 | Unknown [*Oryza sativa*] | 9.0E-06 |
| cln-MIR395 | Unigene15580 | 3 | Aluminum-activated malate transporter [*Glycine max*] | 1.0E-90 |
|  | Unigene27717 | 3 | Peroxidase [*Zea mays*] | 8.0E-11 |
|  | Unigene4333 | 2.5 | Unknown |  |
|  | Unigene54250 | 3 | Rudimentary enhancer [*Glycine max*] | 1.0E-40 |
|  | Unigene56513 | 3 | 1-hydroxy-2-methyl-2-(E)-butenyl 4-diphosphate reductase type 1 [*Ginkgo biloba*] | 2.0E-87 |
|  | Unigene5846 | 3 | SETH1; phosphatidylinositol N-acetylglucosaminyltransferase/ transferase [*Arabidopsis thaliana*] | 2.0E-68 |
|  | Unigene8107 | 3 | Zinc finger (CCCH-type) family protein [*Arabidopsis thaliana*] | 7.0E-75 |
|  | Unigene59613 | 3 | RNA recognition motif (RRM)-containing protein [*Arabidopsis thaliana*] | 0.0E+00 |
| cln-MIR396 | Unigene19040 | 3 | LRR receptor-like serine/threonine-protein kinase FEI 1 [*Arabidopsis thaliana*] | 8.0E-52 |
|  | Unigene13732 | 3 | Unknown |  |
|  | Unigene34726 | 3 | Unknown |  |
|  | Unigene36297 | 3 | Unknown |  |
|  | Unigene42747 | 3 | Unknown |  |
|  | Unigene43805 | 2.5 | Unknown |  |
|  | Unigene4497 | 2 | UPA17 [*Capsicum annuum*] | 2.0E-34 |
|  | Unigene47281 | 3 | FTSH3 [*Oryza sativa*] | 7.0E-50 |
|  | Unigene49408 | 3 | Cysteine protease [*Gossypium hirsutum*] | 2.0E-40 |
|  | Unigene52931 | 3 | S-RNase-binding protein [*Petunia integrifolia*] | 5.0E-16 |
|  | Unigene57122 | 3 | Hydrolase, alpha/beta fold family protein [*Arabidopsis thaliana*] | 2.0E-70 |
|  | Unigene9403 |  | Unknown |  |
|  | Unigene59653 | 2 | ARF1-binding protein [*Arabidopsis thaliana*] | 0.0E+00 |
| cln-MIR398 | Unigene56712 | 2.5 | Pollen-specific protein SF3 [*Helianthus annuus*] | 2.0E-74 |
| cln-MIR399 | Unigene4192 | 3 | SNF2 family DNA-dependent ATPase [*Physcomitrella patens*] | 0.0E+00 |
|  | Unigene12898 | 1.5 | Unknown |  |
|  | Unigene16475 | 3 | Pantothenate kinase [*Arabidopsis thaliana*] | 3.0E-06 |
|  | Unigene17351 | 3 | Unknown |  |
|  | Unigene30681 | 3 | Unknown |  |
|  | Unigene4192 | 3 | SNF2 family DNA-dependent ATPase [*Physcomitrella patens*] | 0.0E+00 |
|  | Unigene51359 | 1.5 | Unknown |  |
|  | Unigene40295 | 3 | Nucleoporin family protein [*Arabidopsis thaliana*] | 1.0E-07 |
| cln-MIR400 | Unigene49313 | 2 | Pentatricopeptide repeat-containing protein [*Arabidopsis thaliana*] | 5.0E-42 |
|  | Unigene9965 | 3 | Beta subunit of rab geranylgeranyltransferase [*Arabidopsis lyrata*] | 1.0E-26 |
|  | Unigene53642 | 3 | Pentatricopeptide repeat-containing protein [*Arabidopsis lyrata*] | 4.0E-16 |
|  | Unigene54767 | 3 | SKIP interacting protein 14 [*Oryza sativa*] | 2.0E-13 |
| cln-MIR403 | Unigene15909 | 2.5 | Tubulin-tyrosine ligase family protein [*Oryza sativa*] | 0.0E+00 |
|  | Unigene57482 | 3 | Unknown |  |
|  | Unigene8297 | 3 | DCN1-like protein 4 [*Zea mays*] | 1.0E-49 |
| cln-MIR408 | Unigene57297 | 2 | Blue copper protein [*Zea mays*] | 3.0E-37 |
|  | Unigene12518 | 3 | Unknown |  |
|  | Unigene16261 | 3 | Unknown |  |
|  | Unigene21319 | 1 | Unknown |  |
|  | Unigene39895 | 3 | Unknown |  |
|  | Unigene59324 | 2.5 | Early-responsive to dehydration protein-related / ERD protein-related [*Arabidopsis thaliana*] | 0.0E+00 |
|  | Unigene54918 | 3 | Queuine tRNA-ribosyltransferase [Phytophthora infestans] | 6.0E-70 |
| cln-MIR528 | Unigene1167 | 3 | SYM10 protein [*Pisum sativum*] | 2.0E-55 |
|  | Unigene16603 | 2.5 | Exonuclease [*Arabidopsis lyrata*] | 1.0E-83 |
|  | Unigene1662 | 2.5 | Glutamate-gated kainate-type ion channel receptor subunit GluR5 [*Populus trichocarpa*] | 1.0E-116 |
|  | Unigene16621 | 3 | Unknown |  |
|  | Unigene16823 | 3 | Pentatricopeptide (PPR) repeat-containing protein [*Arabidopsis thaliana*] | 5.0E-78 |
|  | Unigene2825 | 3 | Cellulose synthase-like C1-2, glycosyltransferase family 2 protein [*Selaginella moellendorffii*] | 0.0E+00 |
|  | Unigene38209 |  | Unknown |  |
|  | Unigene42701 | 3 | GUN5 (GENOMES UNCOUPLED 5); magnesium chelatase [*Arabidopsis thaliana*] | 1.0E-44 |
|  | Unigene48837 | 3 | Short-chain dehydrogenase/reductase family protein [*Arabidopsis lyrata*] | 1.0E-31 |
|  | Unigene55031 | 3 | DNAJ heat shock family protein [*Arabidopsis lyrata*] | 2.0E-66 |
|  | Unigene55455 | 3 | Steroleosin-B [*Sesamum indicum*] | 4.0E-35 |
|  | Unigene56174 | 3 | Unknown |  |
|  | Unigene59569 | 3 | Myosin heavy chain-like [*Oryza sativa*] | 1.0E-150 |
|  | Unigene59574 | 3 | T7 bacteriophage-type single subunit RNA polymerase [*Oryza sativa*] | 0.0E+00 |
| cln-MIR824 | Unigene17658 |  | Unknown |  |
| cln-MIR827 | Unigene36068 | 2.5 | Unknown [*Arabidopsis lyrata*] | 1.0E-20 |
|  | Unigene14600 | 2.5 | ATK1 (*ARABIDOPSIS THALIANA* KINESIN 1); microtubule motor/ minus-end-directed microtubule motor [*Arabidopsis thaliana*] | 1.0E-151 |
| cln-MIR828 | Unigene2867 | 2 | GHMYB10 [*Gossypium hirsutum*] | 2.0E-37 |
|  | Unigene11069 | 3 | Leucine-rich repeat family protein [*Arabidopsis lyrata*] | 1.0E-07 |
|  | Unigene13302 |  | Unknown [*Picea sitchensis*] | 6.0E-12 |
|  | Unigene23430 | 2.5 | Ribosomal protein L13a [*Triticum aestivum*] | 4.0E-07 |
|  | Unigene2358 | 3 | Catalytic/ transferase [*Arabidopsis thaliana*] | 1.0E-87 |
|  | Unigene41790 | 0.5 | Unknown |  |
|  | Unigene59086 | 3 | Protein phosphatase 2A catalytic subunit [*Solanum lycopersicum*] | 1.0E-173 |
| cln-MIR845 | Unigene7408 | 2.5 | bZIP transcription factor [*Phalaenopsis amabilis*] | 1.0E-122 |
|  | Unigene12016 | 2.5 | Unknown |  |
|  | Unigene58482 | 3 | Tryptophan synthase beta subunit [*Camptotheca acuminata*] | 1.0E-169 |
| cln-MIR858 | Unigene10388 | 3 | Unknown |  |
|  | Unigene13575 | 1.5 | Tamyb10-D [*Triticum aestivum*] | 3.0E-51 |
|  | Unigene15489 | 2.5 | R2R3-MYB transcription factor MYB5 [*Picea glauca*] | 2.0E-61 |
|  | Unigene22248 | 2 | Transcription factor DcMYB5 [*Daucus carota*] | 3.0E-22 |
|  | Unigene42567 | 2.5 | R2R3 Myb4b C2 repressor motif protein [*Vitis vinifera*] | 2.0E-51 |
|  | Unigene56574 | 3 | Pseudo-response regulator [*Hordeum vulgare*] | 6.0E-24 |
|  | Unigene58181 | 2.5 | R2R3 Myb14 transcription factor [*Vitis vinifera*] | 1.0E-41 |
|  | Unigene6437 | 3 | R2R3-MYB transcriptional factor [*Gentiana triflora*] | 7.0E-27 |
|  | Unigene8941 | 3 | Cdc2MsC [*Medicago sativa*] | 0.0E+00 |
| cln-MIR2111 | Unigene14713 | 3 | Condensation domain-containing protein [*Arabidopsis lyrata*] | 8.0E-16 |
|  | Unigene18876 | 1 | F-box family protein [*Populus trichocarpa*] | 1.0E-108 |
|  | Unigene3305 | 1.5 | Unknown [*Oryza sativa*] | 2.0E-10 |
|  | Unigene56676 | 1 | Transport protein particle (TRAPP) component Bet3 family protein [*Arabidopsis thaliana*] | 2.0E-94 |
| cln-MIR5083 | Unigene13387 | 3 | Unknown [*Oryza sativa*] | 2.0E-11 |
| cln-MIRn1 | Unigene31829 | 3 | Glycosyltransferase [*Lycium barbarum*] | 4e-12 |
|  | Unigene12209 | 3 | Cl-channel clc-7 [*Populus trichocarpa*] | 0.0 |
|  | Unigene5449 | 1.5 | Unknown |  |
|  | Unigene16369 | 2.5 | Unknown |  |
