# Supplementary material for: Identification and characterization of small non-coding RNAs from Chinese fir by high throughput sequencing
Source: BMC Plant Biol. 2012 Aug 15;12:146. doi: 10.1186/1471-2229-12-146 (PMC3462689; doi:10.1186/1471-2229-12-146)
Supplement: Additional file 8 — Dicer-like proteins in Chinese fir. [file 1471-2229-12-146-S8.doc]

**Additional file 8 Dicer-like proteins in Chinese fir.**

| **Unigene sequence** | **Domain name** | | | | | | | | |
| --- | --- | --- | --- | --- | --- | --- | --- | --- | --- |
|  | **DExD** | **Helicase-C** | **Duf283** | **PAZ** | **RNase III** | **dsRBD** | **Annotation** | **Length (aa)** | **Conserved in other plants** |
| Unigene18579 |  |  |  | + | + | + | DCL1 | 690 | *Oryza sativa* |
| Unigene5851 |  | + |  |  |  |  | DCL1 | 83 | *Physcomitrella patens* |
| Unigene29012 |  |  |  |  | + |  | DCL2 | 62 | *Arabidopsis thaliana* |
| Unigene34518 |  |  |  |  |  |  | DCL3a | 71 | *Oryza sativa* |
| Unigene35698 |  |  |  |  |  |  | DCL3 | 54 | *Physcomitrella patens* |
| Unigene37455 |  |  |  |  |  |  | DCL3 | 76 | *Populus trichocarpa* |
| Unigene16720 |  |  |  |  | + |  | DCL4 | 208 | *Oryza sativa* |
| Unigene3103 |  |  |  |  |  | + | DCL4 | 37 | *Arabidopsis thaliana* |
| Unigene44956 |  |  |  | + |  |  | DCL4 | 91 | *Arabidopsis thaliana* |
| Unigene41759 |  |  |  |  |  |  | DCL4 | 49 | *Arabidopsis thaliana* |
| Unigene11609 |  |  |  |  |  | + | Dicer-like | 92 | *Oryza sativa* |
| Unigene16794 |  |  |  |  | + |  | Dicer-like | 193 | *Populus trichocarpa* |
| Unigene25523 | + |  |  |  |  |  | Dicer-like | 58 | *Populus trichocarpa* |
| Unigene40285 |  |  |  |  | + |  | Dicer-like | 81 | *Oryza sativa* |
| Unigene40957 |  |  |  | + |  |  | Dicer-like | 93 | *Physcomitrella patens* |
| Unigene44442 |  |  |  |  | + |  | Dicer-like | 106 | *Populus trichocarpa* |
| Unigene4521 |  | + |  |  |  | + | Dicer-like | 150 | *Populus trichocarpa* |
| Unigene45244 |  |  |  |  |  | + | Dicer-like | 104 | *Populus trichocarpa* |
| Unigene47836 |  |  |  |  | + | + | Dicer-like | 118 | *Populus trichocarpa* |
| Unigene48030 | + |  |  |  |  |  | Dicer-like | 130 | *Medicago truncatula* |
| Unigene48281 |  |  |  |  | + |  | Dicer-like | 133 | *Oryza sativa* |
| Unigene49008 |  | + |  |  |  |  | Dicer-like | 138 | *Populus trichocarpa* |
| Unigene21838 |  |  |  |  |  |  | Dicer-like | 36 | *Physcomitrella patens* |
| Unigene22262 |  |  |  |  |  |  | Dicer-like | 52 | *Populus trichocarpa* |
| Unigene3157 |  |  |  |  |  |  | Dicer-like | 66 | *Populus trichocarpa* |
| Unigene35924 |  |  |  |  |  |  | Dicer-like | 78 | *Populus trichocarpa* |
| Unigene37365 |  |  |  |  |  |  | Dicer-like | 78 | *Medicago truncatula* |
| Unigene51372 |  |  |  |  |  |  | Dicer-like | 165 | *Medicago truncatula* |
| Unigene5165 |  |  |  |  |  |  | Dicer-like | 182 | *Populus trichocarpa* |
