# Supplementary material for: Identification and characterization of small non-coding RNAs from Chinese fir by high throughput sequencing
Source: BMC Plant Biol. 2012 Aug 15;12:146. doi: 10.1186/1471-2229-12-146 (PMC3462689; doi:10.1186/1471-2229-12-146)
Supplement: Additional file 10 — Unigenes involved in the biogenesis and action of 24-nt rasiRNAs in Chinese fir. [file 1471-2229-12-146-S10.doc]

**Additional file 10 Unigenes involved in the biogenesis and action of 24 nt rasiRNAs in Chinese fir.**

| **Gene name** | **Unigene annotation** | **Unigene** | **Conserved in other plants** | **E-score** |
| --- | --- | --- | --- | --- |
| *RNA polymeraseIV* (*Pol IV*) | RNA polymerase IV largest subunit | Unigene15095 | *Glaucocystis nostochinearum* | 5.00E-48 |
|  |  | Unigene15373 | *Arabidopsis thaliana* | 3.00E-20 |
|  |  | Unigene18484 | *Arabidopsis thaliana* | 2.00E-27 |
|  |  | Unigene42235 | *Glaucocystis nostochinearum* | 8.00E-22 |
|  |  | Unigene51177 | *Arabidopsis thaliana* | 7.00E-16 |
|  |  | Unigene38826 | *Glaucocystis nostochinearum* | 5.00E-10 |
|  |  | Unigene55076 | *Arabidopsis thaliana* | 7.00E-06 |
|  |  | Unigene55221 | *Glaucocystis nostochinearum* | 6.00E-16 |
|  |  | Unigene11511 | *Arabidopsis thaliana* | 2.00E-23 |
|  | RNA polymerase IV second largest subunit | Unigene41476 | *Zea mays* | 6.00E-16 |
|  |  | Unigene4279 | *Ginkgo biloba* | 2.00E-23 |
|  |  | Unigene6654 | *Solanum lycopersicum* | 1.00E-16 |
|  |  | Unigene14496 | *Chlamydomonas reinhardtii* | 3.00E-14 |
|  |  | Unigene12438 | *Ginkgo biloba* | 0.00E+00 |
|  |  | Unigene42360 | *Ginkgo biloba* | 6.00E-19 |
|  |  | Unigene32251 | *Rhododendron macrophyllum* | 2.00E-06 |
|  | RNA polymerase IV subunit | Unigene41283 | *Populus trichocarpa* | 6.00E-11 |
|  |  | Unigene1443 | *Populus trichocarpa* | 6.00E-07 |
|  |  | Unigene1969 | *Populus trichocarpa* | 3.00E-21 |
|  |  | Unigene7815 | *Populus trichocarpa* | 2.00E-07 |
|  | Nuclear RNA polymerase D 1A | Unigene32548 | *Arabidopsis thaliana* | 3.00E-12 |
| *RNA-dependent RNA polymerase2* (*RDR2*) | RNA-dependent RNA polymerase 2 | Unigene15236 | *Arabidopsis thaliana* | 5.00E-13 |
|  |  | Unigene15510 | *Arabidopsis thaliana* | 6.00E-27 |
|  |  | Unigene34941 | *Arabidopsis thaliana* | 4.00E-11 |
|  |  | Unigene36863 | *Oryza sativa* | 5.00E-13 |
|  |  | Unigene44711 | *Arabidopsis thaliana* | 6.00E-06 |
|  |  | Unigene46207 | *Arabidopsis thaliana* | 3.00E-36 |
|  |  | Unigene48870 | *Arabidopsis thaliana* | 4.00E-29 |
|  |  | Unigene56340 | *Arabidopsis thaliana* | 1.00E-81 |
|  |  | Unigene2719 | *Arabidopsis thaliana* | 8.00E-06 |
|  |  | Unigene13690 | *Arabidopsis thaliana* | 1.00E-51 |
| *Dicer-like3* (*DCL3*) | DCL3a | Unigene16720 | *Oryza sativa* | 5.00E-55 |
|  |  | Unigene16794 | *Oryza sativa* | 1.00E-39 |
|  |  | Unigene18579 | *Oryza sativa* | 5.00E-98 |
|  |  | Unigene25523 | *Oryza sativa* | 2.00E-10 |
|  |  | Unigene29012 | *Oryza sativa* | 9.00E-10 |
|  |  | Unigene34518 | *Oryza sativa* | 1.00E-12 |
|  |  | Unigene37455 | *Oryza sativa* | 8.00E-08 |
|  |  | Unigene40957 | *Oryza sativa* | 3.00E-08 |
|  |  | Unigene44442 | *Oryza sativa* | 1.00E-16 |
|  |  | Unigene44956 | *Oryza sativa* | 8.00E-09 |
|  |  | Unigene45244 | *Oryza sativa* | 2.00E-19 |
|  |  | Unigene47836 | *Oryza sativa* | 3.00E-19 |
|  |  | Unigene48281 | *Oryza sativa* | 3.00E-34 |
|  |  | Unigene49008 | *Oryza sativa* | 4.00E-24 |
|  |  | Unigene52674 | *Oryza sativa* | 1.00E-22 |
|  |  | Unigene53195 | *Oryza sativa* | 2.00E-19 |
|  |  | Unigene4521 | *Oryza sativa* | 9.00E-25 |
|  |  | Unigene11609 | *Oryza sativa* | 2.00E-08 |
| *HEN1* | HEN1 | Unigene8390 | *Physcomitrella patens* | 2.00E-23 |
| *Argonaute4* (*AGO4*) | AGO4 | Unigene55017 | *Nicotiana benthamiana* | 1.00E-56 |
|  | AGO4 | Unigene12511 | *Arabidopsis thaliana* | 2.00E-61 |
|  | AGO4-1 | Unigene51607 | *Nicotiana benthamiana* | 6.00E-53 |
|  | AGO4-2 | Unigene43 | *Nicotiana benthamiana* | 9.00E-15 |
|  | AGO4-2 | Unigene11333 | *Nicotiana benthamiana* | 5.00E-26 |
|  | AGO4A | Unigene11338 | *Oryza sativa* | 5.00E-48 |
|  | AGO4B | Unigene45153 | *Oryza sativa* | 7.00E-13 |
| *Argonaute6* (*AGO6*) | AGO6 | Unigene43 | *Arabidopsis thaliana* | 2.00E-15 |
|  |  | Unigene45153 | *Arabidopsis thaliana* | 4.00E-17 |
|  |  | Unigene11338 | *Arabidopsis thaliana* | 3.00E-44 |
|  |  | Unigene12511 | *Arabidopsis thaliana* | 3.00E-57 |
