# Supplementary material for: Identification and characterization of small non-coding RNAs from Chinese fir by high throughput sequencing
Source: BMC Plant Biol. 2012 Aug 15;12:146. doi: 10.1186/1471-2229-12-146 (PMC3462689; doi:10.1186/1471-2229-12-146)
Supplement: Additional file 11 — Unigenes involved in the biogenesis and action of tasiRNAs in Chinese fir. [file 1471-2229-12-146-S11.doc]

**Additional file 11 Unigenes involved in the biogenesis and action of tasiRNAs in Chinese fir.**

| **Gene name** | **Unigene annotation** | **Unigene** | **Conserved in other plants** | **E-score** |
| --- | --- | --- | --- | --- |
| *RNA-dependent RNA polymerase6* (*RDR6*) | RDR6 | Unigene27546 | *Arabidopsis thaliana* | 2.00E-09 |
|  |  | Unigene34941 | *Arabidopsis thaliana* | 3.00E-20 |
|  |  | Unigene39653 | *Arabidopsis thaliana* | 2.00E-28 |
|  |  | Unigene41510 | *Arabidopsis thaliana* | 6.00E-24 |
|  |  | Unigene419 | *Arabidopsis thaliana* | 0 |
|  |  | Unigene1520 | *Arabidopsis thaliana* | 3.00E-62 |
|  |  | Unigene43057 | *Arabidopsis thaliana* | 2.00E-08 |
|  |  | Unigene4129 | *Arabidopsis thaliana* | 1.00E-49 |
|  |  | Unigene5963 | *Arabidopsis thaliana* | 5.00E-20 |
|  |  | Unigene7625 | *Arabidopsis thaliana* | 2.00E-06 |
|  |  | Unigene10467 | *Arabidopsis thaliana* | 3.00E-22 |
| *Dicer like4* (*DCL4*) | DCL4 | Unigene16720 | *Arabidopsis thaliana* | 1.00E-100 |
|  |  | Unigene16794 | *Arabidopsis thaliana* | 2.00E-39 |
|  |  | Unigene22262 | *Arabidopsis thaliana* | 8.00E-09 |
|  |  | Unigene25523 | *Arabidopsis thaliana* | 1.00E-08 |
|  |  | Unigene34518 | *Arabidopsis thaliana* | 6.00E-09 |
|  |  | Unigene35924 | *Arabidopsis thaliana* | 5.00E-10 |
|  |  | Unigene41759 | *Arabidopsis thaliana* | 8.00E-11 |
|  |  | Unigene44442 | *Arabidopsis thaliana* | 4.00E-17 |
|  |  | Unigene44956 | *Arabidopsis thaliana* | 1.00E-14 |
|  |  | Unigene49008 | *Arabidopsis thaliana* | 1.00E-24 |
|  |  | Unigene53195 | *Arabidopsis thaliana* | 7.00E-20 |
|  |  | Unigene57801 | *Arabidopsis thaliana* | 6.00E-09 |
|  |  | Unigene3103 | *Arabidopsis thaliana* | 4.00E-09 |
|  |  | Unigene4521 | *Arabidopsis thaliana* | 4.00E-34 |
|  | Endoribonuclease Dicer homolog 4 | Unigene16720 | *Oryza sativa* | 1.00E-105 |
|  |  | Unigene16794 | *Oryza sativa* | 3.00E-41 |
|  |  | Unigene22262 | *Oryza sativa* | 5.00E-10 |
|  |  | Unigene25523 | *Oryza sativa* | 1.00E-10 |
|  |  | Unigene29012 | *Oryza sativa* | 1.00E-09 |
|  |  | Unigene35924 | *Oryza sativa* | 4.00E-10 |
|  |  | Unigene41759 | *Oryza sativa* | 3.00E-09 |
|  |  | Unigene44442 | *Oryza sativa* | 4.00E-18 |
|  |  | Unigene44956 | *Oryza sativa* | 1.00E-13 |
|  |  | Unigene49008 | *Oryza sativa* | 2.00E-25 |
|  |  | Unigene52674 | *Oryza sativa* | 2.00E-14 |
|  |  | Unigene53195 | *Oryza sativa* | 3.00E-18 |
|  |  | Unigene57801 | *Oryza sativa* | 6.00E-17 |
|  |  | Unigene4521 | *Oryza sativa* | 7.00E-35 |
|  |  | Unigene5851 | *Oryza sativa* | 4.00E-11 |
| *Argonaute7* (*AGO7*) | AGO7 | Unigene15418 | *Arabidopsis thaliana* | 9.00E-13 |
|  |  | Unigene16266 | *Arabidopsis thaliana* | 1.00E-126 |
|  |  | Unigene31751 | *Arabidopsis thaliana* | 1.00E-28 |
|  |  | Unigene35230 | *Arabidopsis thaliana* | 3.00E-26 |
|  |  | Unigene50056 | *Arabidopsis thaliana* | 4.00E-35 |
|  |  | Unigene54975 | *Arabidopsis thaliana* | 2.00E-53 |
|  |  | Unigene59409 | *Arabidopsis thaliana* | 4.00E-73 |
|  | AGO7 | Unigene15418 | *Oryza sativa* | 6.00E-24 |
|  |  | Unigene16266 | *Oryza sativa* | 1.00E-126 |
|  |  | Unigene31751 | *Oryza sativa* | 1.00E-29 |
|  |  | Unigene35230 | *Oryza sativa* | 2.00E-25 |
|  |  | Unigene37458 | *Oryza sativa* | 5.00E-08 |
|  |  | Unigene50056 | *Oryza sativa* | 3.00E-33 |
|  |  | Unigene54975 | *Oryza sativa* | 6.00E-52 |
|  |  | Unigene59409 | *Oryza sativa* | 9.00E-90 |
