# Supplementary material for: Identification and characterization of small non-coding RNAs from Chinese fir by high throughput sequencing
Source: BMC Plant Biol. 2012 Aug 15;12:146. doi: 10.1186/1471-2229-12-146 (PMC3462689; doi:10.1186/1471-2229-12-146)
Supplement: Additional file 13 — Primers for RT-PCR, qRT-PCR and 5' RACE. [file 1471-2229-12-146-S13.doc]

**Additional file 13 Primers for RT-PCR, qRT-PCR and 5' RACE.**

| **Primer** | **Sequence (5'-3')** |
| --- | --- |
| 5.8s rRNA | CGTCTGCTTGGGCGTCGCACAA |
| Cln-miR156a | CCGTTGACAGAAGATAGAGAGCAC |
| Cln-miR157a | CCTGACAGAAGAGAGTGAGCACAA |
| Cln-miR158a | GGTTTCCAAATGTAGACAAAGCA |
| Cln-miR161a | TTGAAAGTGACTACATCGGGGAA |
| Cln-miR164a | TGGAGAAGCAGGGCACGTGCA |
| Cln-miR164b | TGGAGAAGCAGGGCACGTGCG |
| Cln-miR165a | TCGGACCAGGCTTCATCCCCCAA |
| Cln-miR166a | TCGGACCAGGCTTCATTCCCCAA |
| Cln-miR168a | TCGCTTGGTGCAGGTCGGGAA |
| Cln-miR169a | TGAGCCAAAGATGACTTGCCGAA |
| Cln-miR171a | TTGAGCCGTGCCAATATCTCT |
| Cln-miR172a | GAGAATCTTGATGATGCTGCAT |
| Cln-miR390a | AAGCTCAGGAGGGATAGCGCCAA |
| Cln-miR408a | ATGCACTGCCTCTTCCCTGGCAA |
| Cln-miR824 | TAGACCATTTGTGAGAAGG |
| Cln-miR894 | GTTTCACGTCGGGTTCACC |
| Cln-miRn1 | TGGCATCTGTCGAGGTCATCTA |
| Cln-tasiR2142 | TTCTTGACCTTGTAAGACCCCAA |
| Cln-GAPDHF | TTCACAGACAAAGAAAAGGCCTCT |
| Cln-GAPDHR | TGCTCATTCACACCAACAACAAAC |
| Cln-miR164bF | CCTGGTGGAGAAGCAGGGCACGTGCGA |
| Cln-miR164bR | GGGTATAGCCTGGTGGAGAAGGAAAGC |
| Cln-miRn1F | ATACGGTGAGAGGATCTAGGGTTC |
| Cln-miRn1R | AACATCTCTGAATATGCCTGCAAA |
| Cln-Unigene2872P1 | CACTTCTTCTCTTCATTTTCTTGT |
| Cln-Unigene2872P2 | CGATTCTCTGCAATTAGTTCTCTC |
| Cln-Unigene7992P1 | AATGGTAGTACAACACAGAGGGCT |
| Cln-Unigene7992P2 | ACCCCAAAGCCTGCTTTTAATTGA |
| Cln-Unigene28340P1 | TTCTACCATGCCCATTGCCCCTAA |
| Cln-Unigene28340P2 | TACCATGCCCATTGCCCCTAAGAT |
| Cln-Unigene56583P1 | GCCTCAGCAGGAGTTTTTGTTGTCATC |
| Cln-Unigene56583P2 | TCAGCAGGAGTTTTTGTTGTCATCTGA |
| Cln-Unigene6526P1 | CTTGATTCCTGTTTGACCTTTCCC |
| Cln-Unigene6526P2 | TTTTCCTAACTGCTTTTCCGTTTG |
